# Ecological connectivity of the marine protected area network in the Baltic Sea, Kattegat and Skagerrak: Current knowledge and management needs

**DOI:** 10.1007/s13280-021-01684-x

**Published:** 2021-12-29

**Authors:** Charlotte Berkström, Lovisa Wennerström, Ulf Bergström

**Affiliations:** Department of Aquatic Resources, Swedish University of Agricultural Sciences, Institute of Coastal Research, Skolgatan 6, 742 42 Öregrund, Sweden

**Keywords:** Connectivity, Dispersal, Ecological coherence, Migration, Marine protected areas (MPAs), Mobile links

## Abstract

Marine protected areas (MPAs) have become a key component of conservation and fisheries management to alleviate anthropogenic pressures. For MPA networks to efficiently promote persistence and recovery of populations, ecological connectivity, i.e. dispersal and movement of organisms and material across ecosystems, needs to be taken into account. To improve the ecological coherence of MPA networks, there is hence a need to evaluate the connectivity of species spreading through active migration and passive dispersal. We reviewed knowledge on ecological connectivity in the Baltic Sea, Kattegat and Skagerrak in the northeast Atlantic and present available information on species-specific dispersal and migration distances. Studies on genetic connectivity are summarised and discussed in relation to dispersal-based analyses. Threats to ecological connectivity, limiting dispersal of populations and lowering the resilience to environmental change, were examined. Additionally, a review of studies evaluating the ecological coherence of MPA networks in the Baltic Sea, Kattegat and Skagerrak was performed, and suggestions for future evaluations to meet management needs are presented.

**Supplementary Information:**

The online version contains supplementary material available at 10.1007/s13280-021-01684-x.

## Introduction

Ecological connectivity promotes persistence and recovery of marine flora and fauna by the dispersal and movement of organisms and material across populations, communities and ecosystems (Balbar and Metaxas 2019). Connectivity may, however, also promote spread and range shifts of species that invade new areas with negative effects on native ecosystems (Holopainen et al. 2016). Movement and dispersal of eggs, spores, larvae and older individuals among spatially distinct entities is often referred to as ecological spatial connectivity (Carr et al. 2017; hereafter connectivity) and is highlighted as an important element in the design of ecologically coherent networks of marine protected areas (MPAs) (Balbar and Metaxas 2019). MPAs have become a key component of conservation and fisheries management and is recognised as a primary management approach in attempts to alleviate anthropogenic pressures and ensure sustainable use of marine resources (Lubchenco et al. 2003; Halpern et al. 2010). MPAs can also play a vital role in climate change adaptation by enhancing ecosystem resilience and protecting vital ecosystem services (Micheli et al. 2012; Carr et al. 2017). MPAs with fishing restrictions may further enhance density, biomass and body size of targeted fish species and restore ecosystem structure and function (Lester et al. 2009; Baskett and Barnett 2015). MPA size and placement are, however, considered critical elements affecting the success of MPAs, as is the connectivity between the individual MPAs in a network (Claudet et al. 2008; Molloy et al. 2009; Vandeperre et al. 2011). The rate of MPA establishment is increasing worldwide as a response to the 2004 decision by the UN Convention on Biological Diversity to achieve effective protection of 10% of marine ecoregions and that MPAs should be *ecologically representative* and *well connected*. Additionally, a resolution for a new global target of 30% protection was adopted by IUCN in 2016, and is also a central part of the new EU Biodiversity Strategy for 2030 (European Commission 2020). For an efficient expansion of the MPA network in Europe and worldwide, there is a need to evaluate the ecological coherence, including aspects of connectivity and representation of crucial habitats and species. Evaluating ecological coherence of MPAs and MPA networks is, however, still in its infancy and information on connectivity has so far rarely been used in the design and development of MPA networks.

There is no set definition for ecological coherence, although a number of criteria can be quantified during assessment; (1) *adequacy*, (2) *representativity*, (3) *replication*, and (4) *connectivity* (Ardron 2008; Sundblad et al. 2011). Adequacy means that the MPA should be of appropriate size, shape and location to maintain ecosystem functions and services. Representativity reflects the proportion of each conservation feature, while replication reflects the number of each conservation feature being protected. Connectivity, in turn, refers to the spatial configuration of the MPA network and the potential for organisms to move between the individual MPAs and other suitable habitats outside the MPA network to maintain functioning meta-populations. In some cases, only habitat within MPAs are considered when evaluating connectivity, the so-called scorched-earth-scenario (Allison et al. 1998; Jonsson et al. 2020). However, viable habitats outside the MPA network may act as stepping-stones for dispersal where MPAs are part of a wider meta-population. Connectivity may also involve the movement between habitat patches within an MPA during various life stages (ontogenetic migrations). It filters through all the above criteria since dispersal or these active migrations will also affect what size, shape and location of an MPA is required in order to protect the species, as well as which habitats to include and in what proportion. This highlights the growing need to evaluate the ecological coherence of MPA networks based on connectivity via active migration and passive dispersal by a range of species (Virtanen et al. 2018; Jonsson et al. 2020). We have reviewed current literature (grey and white) on ecological connectivity and ecological coherence of the MPA network in the Baltic Sea, Skagerrak and Kattegat. The Baltic Sea is a particularly interesting regional sea regarding future management actions because many pressures and impacts are exceptionally severe. At the same time, these pressures are targeted by an internationally advanced governance and management in order to mitigate negative effects, which is relevant also for other regions where international cooperation is needed to meet environmental challenges. The Baltic Sea also stands out in providing accessibility to long-term data series and a strong scientific foundation, providing a unique opportunity to assess the efficacy of management actions (Reusch et al. 2018).

## Methods

In order to summarise information on ecological connectivity in the Baltic Sea and studies testing the ecological coherence of the MPA network, searches were done in Web of Science with a combination of words including: *connectivity, dispersal, home range, fish migrations, nursery, spawning, tagging, and ecological coherence* (search strings in Appendix S1). Relevant home pages and databases including governmental, regional authorities, NGOs and universities were also scanned in order to find reports and grey literature and researchers with relevant knowledge from the study region were contacted to identify additional literature on connectivity. Information was summarised based on species-specific distance measures for:

1. active migrations (home ranges),
2. passive dispersal of eggs, larvae, spores and/or,
3. distribution ranges based on genetic studies.

In cases where maps on dispersal were available, but no distances were stated, we measured approximate maximum distances for larval dispersal using the Google Earth geographical information system, based on models and maps from Hinrichsen et al. (2017b) and Florin et al. (2013), which included maps for Atlantic cod (*Gadus morhua*), European flounder (*Platichtys flesus*) and turbot (*Scophthalmus maximus*).

In order to search for genetic connectivity the search string “genetic* AND (differen* OR structure OR divergen*) AND (Baltic Sea)” were used. Measures on connectivity, migration distances or distribution ranges were identified. In studies where the authors stated a distance at which migration or gene flow occurs or where populations significantly differ, this distance was set as the maximum range. In cases where distribution ranges or differentiation among populations were described in figures or tables, maximum distances of population distribution were measured in Google Earth.

## Active migrations and passive dispersal

**Fig. 1:**
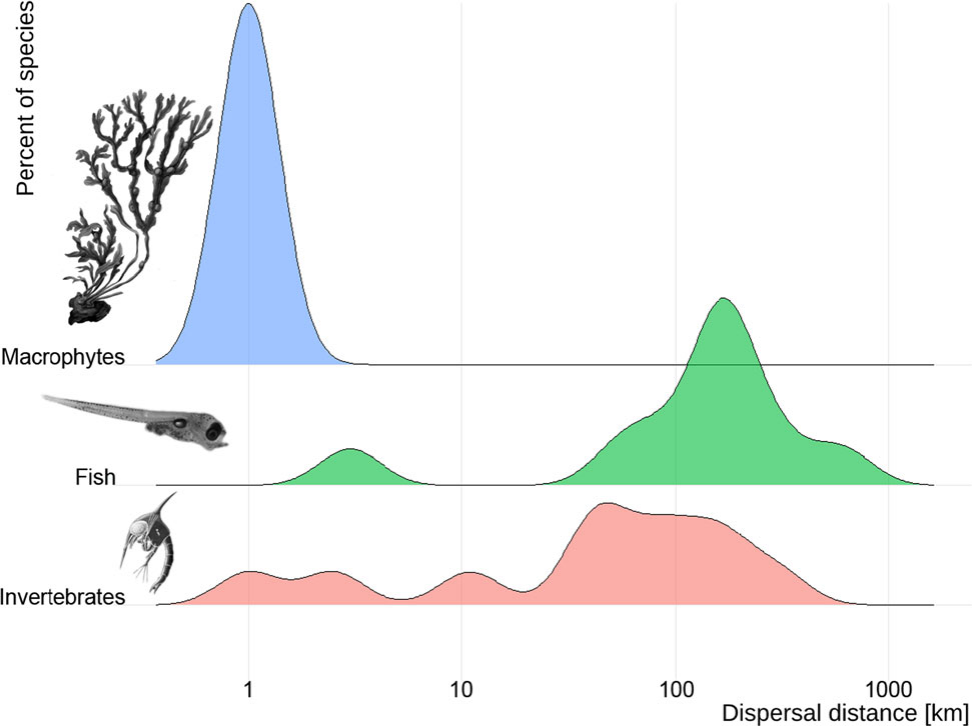
Dispersal distances for spores and pelagic larvae from macropyhytes, invertebrates and fishes in the Baltic Sea, Kattegat and Skagerrak. Distances are maximum distances derived from Table 2, a summary of distances derived from a literature review. Information on dispersal of species with short pelagic stages is limited and hence the number of species with short larval dispersal distances is likely underestimated in the figure. Figure adapted from Gaines et al. (2007)

Connectivity in aquatic environments can be maintained either by active migrations of adult and juvenile organisms or by passive dispersal by currents of eggs, larvae, spores, seeds and fragments. Macrophytes, macroalgae and invertebrates may also disperse by attaching themselves to floating objects (Källström et al. 2008; Winston 2012). Many fish and invertebrates have a pelagic larval phase, spending several weeks in the pelagic zone, and thereby dispersing ten- to hundreds of kilometres (Fig. 1; Kinlan and Gaines 2003). For example, the females of edible crabs *Cancer pagurus* can migrate 100 km upstream to spawn and consequently larvae disperse more than 100 km downstream along the females migration path (Ungfors et al. 2007). Some invertebrate species, however, like the polychaete *Hediste diversicolor* lack a pelagic larval phase and hence have very limited dispersal of only a few metres (Einfeldt et al. 2014).

Macrophyte and macroalgal seeds and spores generally have a more limited dispersal range (< 10 m) than fish and invertebrate larvae (Fig. 1, Table 1). Seeds and spores from sexual reproduction are often heavy and sink within meters of the mother plant, limiting dispersal. However, in some cases, parts of the algae may break off and float long distances before attaching to the bottom in areas with optimal conditions (Tatarenkov et al. 2005). This asexual strategy is a way for algae to increase dispersal and can be found in e.g. bladderwrack, *Fucus vesiculosus* (Rothäusler et al. 2015) and its sister species *F. radicans*, endemic to the Baltic Sea. In fact, this is the main dispersal strategy for *F. radicans* living on the border of its distribution range, which is reflected in its genetic composition where most plants belong to a single clone (Ardehed et al. 2016). In some cases, reproductively mature thalli can also break off and release their gametes in a new location, with potential for long-distance gene flow. Shoots with seeds that have naturally detached from eelgrass (*Zostera marina*) have also been found to float for months, during seed maturation, covering large distances (> 100 km) in Kattegat-Skagerrak and contributing to long distance dispersal (Jahnke et al. 2018). This type of dispersal is, however, not very common in the Baltic Sea where eelgrass blooms more seldom and may, just like for *F. radicans*, be due to the benefits of asexual reproduction when living in environments close to its physiological limits. However, Martínez-Garcia et al. (2021) recently found that sexual reproduction in eelgrass might be common up to the Bornholm Basin at the southern Swedish coast, where a high percentage of multi-locus genotypes were found. Macrophytes and macroalgae may also disperse far by hitchhiking with fish (Boedeltje et al. 2015), birds (Hattermann et al. 2019) or birds feeding on fish (King et al. 2002; Leeuwen et al. 2017). Intact seeds from a number of macrophytes have been found in the faeces of carp feeding on macrophytes and in faeces from cormorants feeding on herbivorous fish. Live fish embryos have also been found to survive gut passages in waterbirds, providing evidence for bird-mediated dispersal of fish (Lovas-Kiss et al. 2020).

**Table 1:** Summary of distribution and habitat use by species from the Baltic Sea, Kattegat and Skagerrak for which published information on dispersal distances is available. See Table 2 for dispersal distances. G = Gulf of Bothnia, B = Baltic Sea, KS = Kattegat-Skagerrak, C/L = coastal/littoral, B = benthic (> 20 m depth), P = pelagic, A = adult, J = juvenile, S = spawning. Habitat acronyms: SH = shallow, D = deep, S = soft, B = bottom, H = hard, M = with macrophytes and macroalgae, NM = no (without) macrophytes and macroalgae, P = pelagic. For maraena whitefish, (M) refers to marine (coastal) spawning ecotype and (AN) to anadromous ecotype spawning in rivers

Species	Common name	Distribution			Zone			Spawning		A habitat	J habitat	S habitat	S depth (m)
		G	B	KS	C/L	B	P	D	P				
*Abramis brama*	Freshwater bream	1	1		1			1		SHSB	SHSBM	SHSBM	0–1.5
*Acipenser oxyrinchus*	Atlantic Sturgeon	1	1	1	1	1		1		SB	SB	SHHB	10–20
*Ammodytes marinus*	Lesser sand-eel			1	1	1		1		SBNM	SBNM	SHSBNM	0–10
*Amphibalanus improvisus*	Bay barnacle	1	1	1	1				1	SHHBNM	SHHBNM	SHHBNM	–
*Anguilla anguilla*	European eel	1	1	1	1		1			–	SHB	–	–
*Ascidia mentula*	Tunicate			1	1				1	DHBNM	DHBNM	DHBNM	5–150
*Ascophyllum nodosum*	Knotted wrack			1	1					SHHBM	SHHBM	SHHBM	0–2
*Aurelia aurita*	Moon jellyfish		1	1	1		1			P	P	P	–
*Cancer pagurus*	Edible/Brown crab			1	1			1		SHBNM	SHBNM	SHBNM	10–40
*Carcinus maenas*	Eruopean shore crab		1	1	1			1		SHB	SHHB	SHHB	0–30
*Ciona intestinalis*	Vase tunicate			1	1				1	HBNM	HBNM	HBNM	0–500
*Clupea harengus*	Atlantic herring	1	1	1	1		1	1		P	P	SHHBM	0–40
*Coregonus maraena*	Maraena whitefish	1	1	1	1			1		SHB	SHHBNM	SHHBNM	0–15
*Coregonus maraena*	Maraena whitefish	1	1	1	1	1		1		SHB	SHHBNM	SHHBNM	0–5
*Coryphaenoides rupestris*	Roundnose grenadier			1		1			1	P	P	P	400–1000
*Cottus gobio*	Bullhead	1	1		1			1		SHHBNM	SHHBNM	SHHBNM	0–6
*Ctenolabrus rupestris*	Goldsinny wrasse			1	1				1	SHHBM	SHHBM	SHHBM	0–20
*Cyclopterus lumpus*	Lumpfish		1	1	1	1		1		HBNM	HBNM	DHBNM	5–40
*Esox lucius*	Pike	1	1	1	1			1		SHB	SHSBM	SHSBM	0–6
*Fucus radicans*	Baltic bladderwrack		1		1					SHHBM	SHHBM	SHHBM	–
*Fucus serratus*	Toothed/Serrated wrack		1	1	1					SHHBM	SHHBM	SHHBM	0–10
*Fucus vesiculosus*	Bladderwrack		1	1	1					SHHBM	SHHBM	SHHBM	0–10
*Gadus morhua*	Atlantic cod		1	1	1	1	1		1	P	SHSBM	P	10–270
*Gasterosteus aculeatus*	Three-spined stickleback	1	1	1	1		1	1		P	SHBM	SHHBM	0–6
*Gobius niger*	Black goby	1	1	1	1			1		SHB	SHB	SHHBNM	0–75
*Gymnocephalus cernua*	Ruffe	1	1		1	1		1		SHSB	–	SHHBM	3–6
*Hediste diversicolor*	Ragworm		1	1	1			1		DSBNM	DSBNM	DSBNM	–
*Homarus gammarus*	European lobster			1	1			1		DHBNM	DHBNM	DHBNM	< 40
*Idotea balthica*	Baltic isopod	1	1	1	1					SHB	SHB	SHB	0–34
*Labrus bergylta*	Ballan wrasse			1	1			1		SHHB	SHHB	SHHBNM	0–30
*Leuciscus idus*	Ide	1	1		1			1		SHB	SHSBM	SHSBM	0–6
*Liparis liparis*	Striped seasnail	1	1	1		1		1		DB	DB	DHBNM	5–300
*Littorina fabalis*	Flat periwinkle			1	1			1		SHHB	SHHBNM	SHHBNM	0–5
*Littorina littorea*	Common periwinkle			1	1				1	SHHB	SHHBNM	SHHBNM	0–15
*Littorina saxatilis*	Rough periwinkle			1	1			1		SHHB	SHHBNM	SHHBNM	0–1
*Lophelia pertusa*	Spider hazards			1	1					DSBNM	DSBNM	DSBNM	80–500
*Lota lota*	Burbot	1	1	1	1			1		SHHB	SHHB	SHHBNM	0.5–3
*Merluccius merluccius*	European hake			1		1			1	P	P	P	100–1000
*Modiolus modiolus*	Northern horsemussel			1	1				1	DHBNM	DHBNM	DSBNM	20–50
*Mytilus edulis*	Blue mussel	1	1	1	1				1	SHHBNM	SHHBNM	SHHBNM	0–10
*Nerophis ophidion*	Straightnose pipefish				1					SHSBM	SHSBM	SHSBM	2–5
*Ostrea edulis*	Flat oyster			1	1				1	SHHBM	SHHBNM	SHHBNM	2–10
*Perca fluviatilis*	European perch	1	1		1			1		SHB	SHSBM	SHSBM	0–5
*Pholis gunnellus*	Rock gunnel	1	1	1	1			1		SHHB	SHHB	SHHBNM	2–6
*Platichthys flesus*	European flounder		1	1	1	1			1	SHB	SHSBMF	DSBNM	0–100
*Platichthys solemdali*	European flounder		1		1	1		1		SHB	SHSBMF	SHSBMF	0–100
*Pleuronectes platessa*	European plaice		1	1	1	1			1	SHSBMF	SHSBMF	SHSBMF	20–90
*Pomatoschistus minutus*	Sand goby	1	1	1	1			1		SHB	SHB	SHSBMF	0–3
*Pygospio elegans*	Polychaete		1	1	1					DSBNM	DSBNM	DSBNM	–
*Ruppia maritima*	Beaked tasselweed		1	1	1					SHSBM	SHSBM	SHSBM	–
*Ruppia spiralis*	Widgeongrass		1	1	1					SHSBM	SHSBM	SHSBM	–
*Rutilus rutilus*	Roach	1	1		1			1		B/P	B/P	SHSBM	0–1
*Salmo salar*	Atlantic salmon	1	1	1	1		1	1		P	SHHBNM	SHHBNM	0.3–3
*Salmo trutta*	Sea trout	1	1	1	1		1	1		P	SHHBNM	SHHBNM	0.3–1
*Sander lucioperca*	Pike-perch	1	1		1			1		SHB	SHSBM	SHSBNM	1–6
*Scomber scombrus*	Atlantic mackerel	1	1	1			1		1	P	P	P	0–20
*Scophthalmus maximus*	Turbot		1	1	1	1		1		SHB	SHSBNM	SHSBNM	0–20
*Skeletonema marioni*	Diatom	1	1	1			1			P	P	P	–
*Solea solea*	Common sole			1	1	1		1		SHSBMF	SHSBNM	SHSBNM	< 30
*Sprattus sprattus*	European sprat		1	1	1		1		1	P	P	P	0–40
*Symphodus melops*	Corkwing wrasse			1	1			1		SHHB	SHHB	SHHBM	0–30
*Zoarces viviparus*	Eelpout	1	1	1	1					SHHB	SHHB	SHHBM	2–20
*Zostera marina*	Eelgrass		1	1	1					SHSBM	SHSBM	SHSBM	0–6
*Zostera noltii*	Dwarf eelgrass			1	1					SHSBM	SHSBM	SHSBM	–

**Fig. 2:**
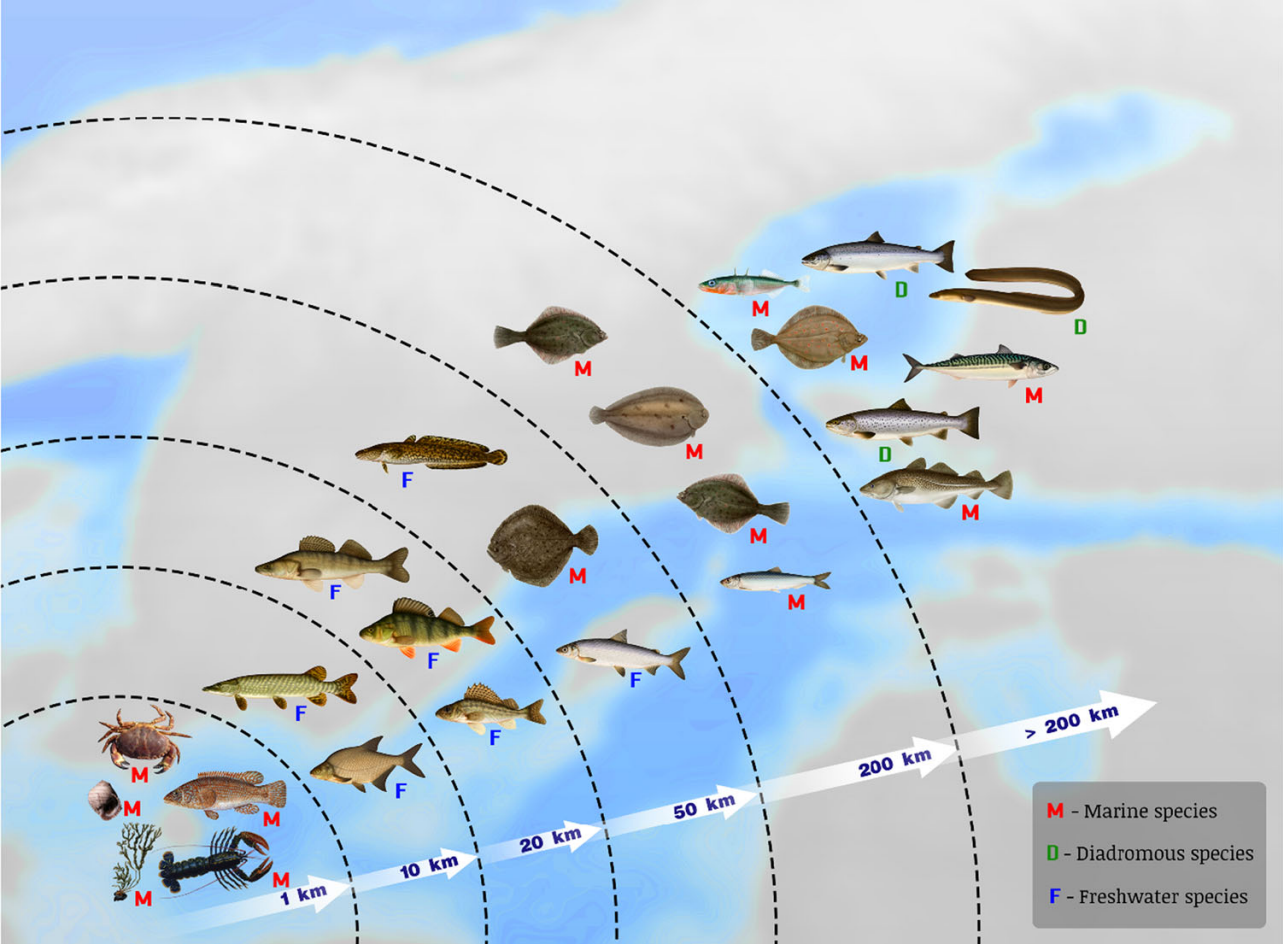
Species have different home ranges based on active migrations and therefore require different sizes and placements of MPAs. MPAs often need to be larger than the species home range to protect adult individuals. The figure illustrates home ranges for some key species found in the Baltic Sea, Kattegat and Skagerrak, for which dispersal distances are known. Species in each home range category are listed from top to bottom: < 1 km *Cancer pagurus*, *Littorina fabalis*, *Labrus bergylta*, *Fucus vesiculosus*, *Homarus gammarus*; < 10 km *Esox lucius Abramis brama*; < 20 km *Sander lucioperca, Perca fluviatilis, Gymnocephalus cernuus*; < 50 km *Zoarces viviparus, Scophthalmus maximus, Coregonus maraena* (sea-spawning); < 200 km *Platichthys flesus, Solea solea, Platichthys solemdali, Clupea harengus*; > 200 km*, Salmo salar, Gasterosteus aculeatus, Anguilla anguilla, Pleuronectes platessa, Scomber scombrus, Salmo trutta and Gadus morhua*. Figure adapted from Green et al. (2014). Illustrations used with permission from ArtDatabanken, Swedish University of Agricultural Sciences

Dispersal by pelagic larvae is more common in marine species compared to freshwater species and is therefore a common feature in Kattegat and Skagerrak while less so in the brackish Baltic Sea, which possesses a unique set of species of both marine and freshwater origin. Roughly 70% of marine invertebrates and the majority of marine fish disperse by larvae (Thorson 1950). Species in the Baltic Sea of marine origin, e.g. cod (*Gadus morhua*), sprat (*Sprattus sprattus*) and flatfish, often spawn in the pelagic zone and have larvae dispersing far with currents during 1–2 months. They also tend to be more mobile as adults with cod migrating up to 1000 km (Table 2). In contrast, species of freshwater origin like pike (*Esox lucius*), perch (*Perca fluviatilis*) and pike-perch (*Sander lucioperca*) tend to spawn closer to the coast in shallow, warm macrophyte habitats during spring where eggs are attached to vegetation or are stationary until they hatch (Table 1). Additionally, these species often have small home ranges, staying close to the coast while marine species tend to migrate long distances (Fig. 2). A reason for this difference is that the Baltic Sea is characterised by strong environmental gradients including temperature and salinity and many species are highly dependent on specific environmental conditions for development and survival during larval- and juvenile stages (Aro 2002).

**Table 2:** Summary of dispersal and migration distances for species in the Gulf of Bothnia, Baltic Proper (including the Gulf of Finland and Riga), Kattegat and Skagerrak displayed as home range (active migrations), dispersal by fragments or larvae/spores/seeds and distributions based on genetic studies. M = Marine and AN = Anadromous. References from which distances have been extracted can be found in Table S1

Species	Common name	Adult homerange	Asexual dispersal		Larval/Propagule	Population
		Mean	Mean	Max	Dispersal	Distribution
*Abramis brama*	Freshwater bream	< 5 km	–	–	–	–
*Acipenser oxyrinchus*	Atlantic Sturgeon	< 800 km	–	–	–	–
*Ammodytes marinus*	Lesser sand-eel	–	–	–	–	300–700 km
*Amphibalanus improvisus*	Bay barnacle	Stationary	–	–	160 km	–
*Anguilla anguilla*	European eel	> 5000 km	–	–	–	> 1000 km
*Ascidia mentula*	Tunicate	Stationary	–	–	1.5 km	–
*Ascophyllum nodosum*	Knotted wrack	Stationary	–	–	> 5 m	–
*Aurelia aurita*	Moon jellyfish	–	–	–	40 km	–
*Cancer pagurus*	Edible/Brown crab	< 1 km	–	–	–	> 1300 km
*Carcinus maenas*	Eruopean shore crab	–	–	–	148–160 km	–
*Ciona intestinalis*	Vase tunicate	–	–	–	–	10 km
*Clupea harengus*	Atlantic herring	150 km	–	–	–	400–1000 km
*Coregonus maraena*	Maraena whitefish (M)	20–40 km	–	–	–	100 km
*Coregonus maraena*	Maraena whitefish (AN)	300–500 km	–	–	–	–
*Coryphaenoides rupestris*	Roundnose grenadier	–	–	–	–	100 km
*Cottus gobio*	Bullhead	–	–	–	160 km	–
*Ctenolabrus rupestris*	Goldsinny wrasse	100 m	–	–	–	–
*Cyclopterus lumpus*	Lumpfish	–	–	–	–	1000 km
*Esox lucius*	Pike	3 km	–	–	–	10–400 km
*Fucus radicans*	Baltic bladderwrack	Stationary	10 m–1 km	> 100 km	1–2 m	550 km
*Fucus serratus*	Toothed/Serrated wrack	Stationary	–	–	1–2 m	2 km
*Fucus vesiculosus*	Bladderwrack	Stationary	10 m–1 km	250 km	1–2 m	10 m–500 km
*Gadus morhua*	Atlantic cod	100–800 km	–	–	600 km	100–400 km
*Gasterosteus aculeatus*	Three-spined stickleback	> 100 km	–	–	–	200–1000 km
*Gobius niger*	Black goby	–	–	–	160 km	–
*Gymnocephalus cernua*	Ruffe	< 15 km	–	–	–	–
*Hediste diversicolor*	Ragworm	–	–	–	Few meters	–
*Homarus gammarus*	European lobster	< 250 m	–	–	–	> 400 km
*Idotea balthica*	Baltic isopod	–	–	–	–	100–300 km
*Labrus bergylta*	Ballan wrasse	100 m	–	–	–	–
*Leuciscus idus*	Ide	–	–	–	–	–
*Liparis liparis*	Striped seasnail	–	–	–	55 km	–
*Littorina fabalis*	Flat periwinkle	2 m	–	–	–	–
*Littorina littorea*	Common periwinkle	–	–	–	300 km	–
*Littorina saxatilis*	Rough periwinkle	2 m	–	–	–	1–2 km
*Lophelia pertusa*	Spider hazards	–	–	–	40 km	< 35 km
*Lota lota*	Burbot	20 km	–	–	–	–
*Merluccius merluccius*	European hake	–	–	–	–	700 km
*Modiolus modiolus*	Northern horsemussel	Stationary	–	–	10 km	–
*Mytilus edulis*	Blue mussel	Stationary	–	–	10–50 km	300–600 km
*Nerophis ophidion*	Straightnose pipefish	–	–	–	160 km	–
*Ostrea edulis*	Flat oyster	Stationary	–	–	88 km	–
*Perca fluviatilis*	European perch	10 km	–	–	0.1–2 km	2–100 km
*Pholis gunnellus*	Rock gunnel	–	–	–	84 km	–
*Platichthys flesus*	European flounder	30–200 km	–	–	300 km	300–600 km
*Platichthys solemdali*	European flounder	30–200 km	–	–	–	300–400 km
*Pleuronectes platessa*	European plaice	300–500 km	–	–	–	200 km
*Pomatoschistus minutus*	Sand goby	–	–	–	160 km	700 km
*Pygospio elegans*	Polychaete	–	–	–	87 km	–
*Ruppia maritima*	Beaked tasselweed	Stationary	–	–	–	4 m–20 km
*Ruppia spiralis*	Widgeongrass	Stationary	5–20 km	179 km	–	20 km
*Rutilus rutilus*	Roach	5 km	–	–	–	–
*Salmo salar*	Atlantic salmon	100–1000 km	–	–	–	50–200 km
*Salmo trutta*	Sea trout	100–300 km	–	–	–	100–200 km
*Sander lucioperca*	Pike-perch	10 km	–	–	–	50–200 km
*Scomber scombrus*	Atlantic mackerel	500 km	–	–	–	–
*Scophthalmus maximus*	Turbot	10–30 km	–	–	200 km	400–1000 km
*Skeletonema marioni*	Diatom	–	–	–	–	20–150 km
*Solea solea*	Common sole	150 km	–	–	–	350–400 km
*Sprattus sprattus*	European sprat	–	–	–	–	150–400 km
*Symphodus melops*	Corkwing wrasse	100 m	–	–	–	60–700 km
*Zoarces viviparus*	Eelpout	–	–	–	–	50–500 km
*Zostera marina*	Eelgrass	Stationary	10–100 km	150–200 km	5 m	300 km
*Zostera noltii*	Dwarf eelgrass	Stationary	–	–	–	65–150 km

Salinity declines in a gradient towards the northern parts of the Baltic Sea, limiting dispersal and survival of marine species and hence acting as dispersal barriers. Temperatures also fluctuate greatly on a yearly basis due to shallower water in the Baltic Sea compared to the deep North Sea (Bekkevold et al. 2015; Berg et al. 2017). These barriers are particularly apparent in the transition region between the North Sea and the Baltic Sea were salinity, depth and currents abruptly change across short distances from marine (35 psu) to brackish (10 psu) conditions (Ulrich et al. 2017), which restricts exchange of organisms between the Baltic and the North Sea (Johannesson and André 2006). This gradient continues throughout the Baltic Sea to the Bothnian Bay furthest north, where salinity conditions are as low as 2–4 psu. The effects of this environmental gradient on the connectivity of common organisms can also be seen in studies on e.g. herring (Jörgensen et al. 2005), sprat (Limborg et al. 2009), perch (Olsson et al. 2011) and whitefish (Olsson et al. 2012b), where genetic patterns in putatively neutral genetic markers differ between areas of different salinity.

Fish generally have larger home ranges than invertebrates, and juveniles generally have smaller home ranges than adults (Fig. 2). Many species, particularly large fish, migrate on a seasonal basis between shallow coastal feeding, spawning and nursery grounds in order to optimise spawning and food intake (Table 2; Aro 1989; Candolin and Voigt 2003; Tibblin et al. 2016). These areas provide optimum conditions for egg and larval development, which are more reliant on higher temperatures than adults. These habitats also provide young with sufficient food and shelter. Seitz et al. (2014) found that as much as 44% of all commercially important species in the northeast Atlantic use shallow coastal areas either as feeding, spawning or nursery areas and that these stocks make up 77% of commercial fish landings. Other species (anadromous species) like salmon (*Salmo salar*) and sea trout (*Salmo trutta*) migrate from feeding grounds in the southern part of the Baltic Sea to rivers in the Bothnian Bay where they spawn (migrating up to 1500 km; Table 2). Although most of the species like pike, perch and various cyprinids spawn in coastal brackish waters, some populations are anadromous and return to freshwater creeks to spawn, similar to salmon and sea trout (Tibblin et al. 2012; Larsson et al. 2015). The European eel (*Anguilla anguilla*) preforms the most extreme spawning migration from freshwater creeks and rivers to the Sargasso Sea, several thousands of kilometres away from the Baltic Sea.

## Genetic connectivity

Genetic connectivity is defined as “the degree to which gene flow affects evolutionary processes within subpopulations” (Lowe and Allendorf 2010). Areas are genetically connected if individuals are dispersed between populations and also contribute genetically to the next generation, i.e. successful reproduction.

Connectivity can be studied using genetic markers in two primary ways. Either indirectly by studies of population structure or directly, identifying putative migrants by inferring the population origin of specific individuals or its parents (Botsford et al. 2009; Planes et al. 2009; Gagnaire et al. 2015). The former is most common, and there are many examples of studies estimating the distribution of populations and/or differentiation among populations in the Baltic Sea, Kattegat, and Skagerrak (Wennerström et al. 2017). The latter example is relatively rare worldwide. Even though genetics is a potentially powerful tool to study connectivity directly, and genetic markers can be used as individual tags, it requires rigorous sampling of target and source populations on a scale that is rarely feasible.

Genetic population range reflects the maximum distance at which populations can be considered connected genetically, either directly through dispersal and following establishment and/or reproduction or indirectly by a multi-generational stepping stone process. Genetic differentiation among populations is affected by a number of processes, such as effective population size and migration distances, while, importantly, our perception of genetic differentiation is affected by the choice of genetic markers (Kinlan and Gaines 2003). Neutral and selected genetic markers for example reflect different processes and genetic significant differences while markers under selection can show genetic differentiation even when gene flow is substantial (e.g. Han et al. 2020). In this study we use the distance at which significant genetic differences are found, regardless of genetic marker studied, as an indication of genetic population range. When compared to other measurements of dispersal, genetic population range can be divided into three groups. Species where the genetic population range is;

1. larger than other measurements of dispersal.
2. about the same as other measurements of dispersal.
3. smaller than other measurements of dispersal.

Species where the genetic population range is larger than other measurements of dispersal include macrophytes, invertebrates, and some species of coastal fish. These species have a sedentary life style with rare long distance dispersal events. In macrophytes, fragments of disconnected plant material can travel long distances (Pereyra et al. 2013). Invertebrates primarily disperse with ocean currents during a planktonic larval stage (Kinlan and Gaines 2003). For coastal fish species like pike and turbot it might be possible for some individuals to migrate over large distances (Laikre et al. 2005; Florin and Franzén 2010; Wennerström et al. 2016). These rare migration events do not affect ecological connectivity and population dynamics greatly, but might have a large impact on genetic patterns.

The second group, where genetic population range and other dispersal measurements are roughly the same, includes marine species like cod, herring, sprat and three-spined stickleback. These species have large population sizes and few barriers to migration, which is reflected in genetic patterns where differences among populations typically are small. Also species with clonal dispersal, such as *Fucus radicans*, have matching ranges of different dispersal measurements.

There are also a number of species where the genetic population range is smaller than other measurements of individual movement. Species with a strong homing behaviour, like salmon and trout belong to this group. These species typically have unique spawning populations in individual streams, or even multiple populations within streams (Koljonen et al. 2002; Vasemägi et al. 2005). During feeding, however, they can migrate hundreds of kilometres. For species like salmon, sea trout and pike, however, it may be a disadvantage to solely use genetic methods to study connectivity since many individuals are farmed and released for conservation purposes. Genetic variation will not always reflect natural dispersal in those cases.

## Incorporating connectivity in management and marine protected area network design

When designing and implementing MPAs it is important to acknowledge the different types of connectivity including active migrations and passive dispersal as well as the movements and needs of different life-stages (Félix-Hackradt et al. 2018). It is also important to separate between typical home ranges and maximum migration distances since home ranges reflect scales relevant for population dynamics while maximum distances are more important for the genetic variation between populations (Bergström et al. 2007). An MPA may either be larger than an organisms’ dispersal range in order to keep a viable population within the MPA or consist of a network of MPAs placed with distances equivalent to organisms’ dispersal ranges in order to connect populations within the network (Carr et al. 2017). Additionally, it should include all habitats needed during an organism’s lifecycle (spawning, nursery and feeding) to make sure the MPA network is ecologically coherent, unless these habitats are found in adequate condition outside the MPA network and within the organism’s dispersal range. Connectivity within MPAs is hence important for species with short dispersal ranges and found in fragmented habitats, while connectivity between MPAs and the surrounding area is important for dispersal and genetic exchange between populations across larger areas (Andersson et al. 2008).

Understanding dispersal patterns between MPAs is complex due to dispersal being dictated by currents, time of year, amount of time larvae/spores spend in the pelagic and at what depth they are located (Kinlan and Gaines 2003). How far larvae/spores disperse is determined by how long they remain viable in the water column and what environmental conditions they are subjected to. Larvae can partly regulate their dispersal by shifting between water layers and hence choose which currents to disperse with (Moksnes et al. 2014a). Larvae with long larval durations that spend time close to the surface generally disperse further than larvae with short larval durations that spend time at deeper depths (> 20 m) since surface currents often are faster than currents below the thermocline (Moksnes et al. 2014b). Sea star- and cod larvae are examples of organisms dispersing far in surface waters while mussels and gastropods often spend time below the thermocline and therefore have more limited dispersal (Moksnes et al. 2014b). Although some larvae have long larval durations, their dispersal may be limited by local water currents such as eddies that retain larvae within a bay or fjord (Cowen et al. 2000; Øresland and Ulmestrand 2013). Local hydrodynamic conditions are therefore equally important to consider in MPA design as is large scale climate and hydrological conditions. In fact, otolith and genetic studies have found that pelagic larvae have more limited dispersals than previously thought, often within 10–100 km (Palumbi 2004; Cowen et al. 2006; Benestan et al. 2021). Fish larvae and larvae of larger decapods like spiny lobster (Palinuridae) may also actively swim against currents in their last stages of development before settling and partly affect dispersal (Fisher 2005; Leis 2006). For instance, Faillettaz et al. (2018) found that fish larvae behaviour affected the dispersal among MPAs in the Mediterranean.

Considering the large environmental changes in the Baltic Sea and the mix of marine and freshwater species, environmental variables including salinity, temperature, oxygen levels and currents will limit dispersal and hence need to be taken into account when deciding on sizes, numbers and placement of MPAs. This poses extra challenges when managing the Baltic Sea area. If MPA design is developed based on species and habitat maps, many of these environmental variables will automatically be incorporated since the variables dictate species ranges. Furthermore, local adaptations particularly prominent in the two endemic species, Baltic flounder *Platichthys solemdali* and the brown algae *Fucus radicans*, result in contrasting dispersal and connectivity patterns in the two different organismal groups (Momigliano et al. 2018). The Baltic flounder has developed heavier eggs, different larval behaviour and spawn in coastal areas allowing shorter dispersal compared to its closest relative European flounder (Nissling et al. 2017; Corell and Nissling 2019). In contrast, *F. radicans* relies on asexual reproduction and disperses farther than its closest relative bladderwrack, and therefore has a more northern distribution in the Bothnian Sea where the population is represented by one dominant clone (Bergström et al. 2005; Tatarenkov et al. 2005; Ardehed et al. 2015).

The northern parts of the Gotland basin, with low oxygen levels, is considered a barrier for adult European flounder, restricting their migration northwards across these unfavourable areas. Their pelagic larvae, on the other hand, may disperse across this hypoxic/anoxic zone (Aro 1989; Florin and Höglund 2008). Furthermore, European flounder and cod need areas with high salinity and oxygen levels for egg and larval survival and are therefore limited to spawn in isolated deep water basins with suitable conditions, such as the Bornholm Basin, the Gdansk Deep, and the Gotland Basin. Limited spawning due to unfavourable oxygen conditions in the Gotland basin may explain the large declines in catches of European flounder close to its geographical boarder in the Gulf of Finland (GoF) since the mid-1980s (Jokinen et al. 2019). Similarly, the spatial contraction of the eastern Baltic cod population to the Bornholm basin has been suggested to be a consequence of a loss of suitable spawning habitat in the Gotland Basin (Bartolino et al. 2017).

Mobility of species differ between species of marine or freshwater origin. Large stretches of deep water may therefore function as barriers for coastal species of freshwater origin (e.g. perch) which are relatively stationary and have short larval dispersal (Olsson et al. 2011). This strategy of a more direct development may be beneficial for species living close to their environmental limits. The proportion of freshwater and marine species also varies between water basins, e.g. freshwater and anadromous species with low dispersal dominate in the Bothnian Bay while marine species with pelagic larval phases and larger dispersal ranges dominate towards the North Sea.

## Considering climate change and other anthropogenic disturbances

An MPA network should ideally withstand local and global disturbances from climate change and other anthropogenic pressures. Including connectivity in MPA design may be even more crucial in the light of climate change because dispersal of organisms between areas will facilitate both survival and recovery of populations (Magris et al. 2014; Balbar and Metaxas 2019). Dispersal and distribution ranges of species may, however, be negatively affected by climate change due to changes in temperature, salinity and water movement (Bruno et al. 2018). Changes in dispersal between MPAs may in turn decrease the network’s ability to withstand environmental changes. When planning additional MPAs it is therefore important to consider their impact on connectivity, and make sure they are placed in locations that will strengthen the connectivity of the network and increase its resilience against future changes (Carr et al. 2017). An MPA network with sufficient connectivity may also allow the range shift of locally adapted genotypes to move with climate change. Wilson et al. (2020) identified a number of climate change adaptation strategies for MPAs including increased resilience, connectivity and heterogeneity, protection of climate refugia and future habitat, reduction of other stressors and increasing adaptive capacity. Additionally, they found that 82% of real-world examples of climate change adaptation in MPA planning derives from tropical reefs, highlighting the need for addressing temperate ecosystems. In Sweden, climate change effects on species distributions are considered in marine spatial planning, and climate refuges for some important species have been mapped in the Baltic Sea (Hammar and Mattson 2017).

Climate change in coastal and pelagic areas is already apparent in the Baltic Sea, Kattegat and Skagerrak with increasing temperatures, shorter ice periods and extended bottoms with hypoxic conditions (Viitasalo 2019). These changes affect species distribution ranges, spawning behaviour and habitat selection and may have both negative and positive effects on populations (Härmä et al. 2008; Olsson et al. 2012a; Nissling and Wallin 2020). For example, the distribution range of cod is moving north on a global scale with increasing temperatures (Werner et al. 2016), while bladderwrack’s distribution range is decreasing in the Baltic Sea following a decrease in salinity and increase in acidity (Jonsson et al. 2018). The disappearance of canopy-forming vegetation, like bladderwrack, on hard substrates is problematic since there are no freshwater plants or algae that can replace the function of this keystone species. The observed decrease in salinity is due to an increase in precipitation and terrestrial runoff. Consequently, the freshwater gradient moves southwards in the Baltic Sea (Wake 2012). Species of freshwater origin may benefit from increased precipitation and a decrease in salinity due to an expansion of spawning and nursery areas in coastal areas (Härmä et al. 2008). Moreover, the shorter winters and higher water temperatures will in turn increase growth rates of species adapted to warm water conditions, e.g. perch, while species adapted to spawning in cold water, like whitefish (*Coregonus maraena*), may instead fail (Veneranta et al. 2013). Also, many coastal spawning and nursery areas will dry out earlier since the increase of water flow takes place earlier on in the season than usual (Larsson et al. 2015). Besides a decrease in salinity, the increase of anoxic conditions due to both temperature increases and eutrophication in the Baltic Sea may have large effects on marine species, like European flounder and cod, which possess larvae that are highly dependent on areas with high salinity and oxygen conditions (Orio et al. 2017, 2019). This increase of bottoms with hypoxic conditions and decrease of spawning grounds will in turn have consequences on dispersal and geographical range. Similarly, habitat shrinkage due to increased temperatures has been observed for cod around Denmark resulting in increased fragmentation and decreased connectivity of viable habitats (Dinesen et al. 2019). Hypoxic or anoxic conditions may also occur in coastal areas with increased temperatures affecting spawning and nursery areas for a number of species (Viitasalo 2019).

Due to many species living close to their physiological salinity limits, most species in the Baltic Sea will be affected and some species may even disappear. Climate effects on populations have also been observed in Kattegat, where Arctic-Boreal species have decreased in abundance and species range while Mediterranean-Boreal species have increased in abundance and species range (Göransson 2017). Arctic-Boreal species also tend to have shorter dispersal ranges than Mediterranean-Boreal species making it more difficult for these species to recolonise areas where abundances have decreased. Additionally, an increase in ocean temperature may decrease planktonic larval duration and hence dispersal distance, justifying the need for larger and closer MPAs within the network in this region (Álvarez-Romero et al. 2018).

Higher temperatures together with eutrophication and overfishing of top predators increase macroalgal blooms, both as drifting algal mats and as epiphytes on large canopy-forming macrophytes and macroalgae like eelgrass and bladderwrack (Cossellu and Nordberg 2010). These canopy forming macrophytes and algae are important spawning and nursery areas for many fish species and might disappear due to macrophytes and macroalgae being smothered and/or shaded by epiphytes (Rönnbäck et al. 2007). The lack of top predators and hence top-down control in Baltic Sea coastal areas has resulted in trophic cascades with an increase of mesopredators like the three-spined stickleback, a reduction in important grazers (stickleback prey) and in increase in epiphytic algae (Donadi et al. 2017; Eklöf et al. 2020). Drifting mats of filamentous algae may also cover large areas of sandy bottoms and limit the amount of nursery area for commercially important flatfish (Pihl et al. 2005). If these key habitats disappear, connectivity by species reliant on these habitats may decrease or cease with negative effects on population dynamics.

Physical disturbance from jetties, dredging and boat traffic can have negative effects on nursery grounds (Macura et al. 2019). Many shallow protected bays with a high density of jetties and intense boat traffic have 40–80% less vegetation than bays with few jetties in the Baltic Sea (Hansen et al. 2018). Also, the diversity of macrophytes are negatively affected by jetties and boat traffic with sensitive species often disappearing (Eriksson et al. 2004; Sandström et al. 2005). These shallow nursery areas are rather rare within the Baltic Sea seascape and are highly sensitive to disturbances (Snickars et al. 2010). Studies have shown a positive relationship between the amount of benthic vegetation and pike, perch and cyprinid larvae in areas where such macrophyte and macroalgal habitats are generally rare (Sundblad and Bergström 2014; Hansen et al. 2018). Negative effects of jetties on eelgrass meadows, also important nursery grounds (Staveley et al. 2016; Perry et al. 2018), was found in Skagerrak and Kattegat (Eriander et al. 2017). To alleviate the effects of physical disturbance and habitat loss, attempts to identify and restore important nursery areas such as coastal wetlands and seagrass beds have been made as a mean to decrease fragmentation and increase connectivity (Nilsson et al. 2014; Eriander et al. 2016; Jahnke et al. 2018, 2020).

## Ecological coherence of the marine protected area network

In total, fifteen studies have evaluated aspects of ecological coherence of the MPA network in the Baltic Sea (10 studies), Kattegat-Skagerrak (4 studies) and both regions (1 study). The first two studies were conducted in the Baltic Sea in 2007 (Bergström et al. 2007; Piekäinen and Korpinen 2007) and the first evaluation in Kattegat-Skagerrak in 2013 (Johnson et al. 2013). Most studies found that the network of MPAs was non-coherent (Table 3). In some cases the network fulfilled one of the four coherence criteria (adequacy, representativity, replication, and connectivity), but far from all criteria. In general the criterion on how well connected the MPA network is, was evaluated. Focus was mainly on larval dispersal using hydrodynamic models, with three studies in Kattegat-Skagerrak (Moksnes et al. 2014b, 2015; Jonsson et al. 2016b), three in the Baltic Sea (Corell et al. 2012; Nilsson Jacobi et al. 2012; Jonsson et al. 2020) and one in both regions (Assis et al. 2021). The models were parameterised using data on drift depths and seasonal recruitment from plankton surveys. Larval dispersal was simulated by releasing virtual larvae with various swimming abilities from areas corresponding to natural distributions. The models provided useful estimates of larval dispersal for coarse-scale analyses. However, the large-scale hydrodynamic models do not account for local conditions in topographically complex areas like archipelagos. This may overestimate the dispersal of some species in coastal regions. Additionally, the models do not account for habitat differences between areas due to lack of comprehensive habitat maps of the Baltic Sea and Kattegat-Skagerrak. Results from these studies still indicate that the network of Natura 2000 areas (Corell et al. 2012; Nilsson Jacobi et al. 2012) and HELCOM MPAs (Jonsson et al. 2020) are non-coherent in the Baltic Sea and that the network is particularly weak along the Swedish east coast and the Finnish west coast. Additionally, the Swedish MPAs are generally too small for larvae spawned within MPAs to be retained and contribute to viable populations within these areas (Jonsson et al. 2020). The OSPAR MPA network was also found to be non-coherent in Kattegat-Skagerrak. The protected areas in the south of Kattegat and Danish Belt are the most important MPAs with regard to maintaining larval connectivity along the Swedish coastline due to surface currents mainly heading north (Moksnes et al. 2014b; Jonsson et al. 2016b), but MPAs are too small and inaccurately placed in order to maintain sufficient connectivity.

**Table 3:** Summary of studies evaluating ecological coherence of MPA networks in the Baltic Sea, Kattegat and Skagerrak

Geografical area	Year	Organism	Species	Type av connectivity	References	Publication	Resultats
Baltic Sea	2007	Macrophytes/Macroalgae/Inverts/Fish	5 key organisms	Larval disp + nursery gounds	Piekäinen and Korpinen (2007)	Report	Partly coherent
Baltic Sea	2007	Fish	Herring	Spawning + nursery grounds	Bergström et al. (2007)	Report	Partly coherent
Baltic Sea	2010	Macrophytes/Macroalgae/Inverts/Fish	5 key organisms	Larval disp + nursery gounds	HELCOM (2010)	Report	Partly coherent
Baltic Sea	2011	Fish	Pike, Perch, Pike-perch, Roach	Nursery grounds	Sundblad et al. (2011)	Article	Not coherent
Baltic Sea	2012	Inverts/Fish	Several	Larval disp	Corell et al. (2012)	Article	Not coherent
Baltic Sea	2012	Inverts	Blue mussel	Larval disp	Nilsson Jacobi et al. (2012)	Article	Not coherent
Baltic Sea	2015	–	–	Larval disp	Wolters et al. (2015)	Report	Not coherent
Baltic Sea	2017	Macrophytes/Macroalgae/Inverts/Fish	5 key organisms	Larval disp + nursery gounds	HELCOM (2016)	Report	Not coherent
Baltic Sea	2018	Macrophytes/Macroalgae/Inverts/Fish	Several	Distribution maps/distance-based connectivity (all life-stages)	Virtanen et al. (2018)	Article	Not coherent
Baltic Sea	2019	Inverts/Fish	Several	Larval disp	Jonsson et al. (2020)	Article	Not coherent
Baltic Sea/Kattegatt/Skagerrak	2021	Macrophytes/Macroalgae/Inverts/Fish	Several	Larval disp	Assis et al. (2021)	Article	Not coherent
Kattegatt/Skagerrak	2013/2014	Macrophytes/Macroalgae/Inverts/Fish	Several	Larval/seed disp + nursery gounds	Johnson et al. (2013, 2014)	Report + Article	Not coherent
Kattegatt/Skagerrak	2014	Inverts/Fish	45 fish + 80 inverts	Larval disp	Moksnes et al. (2014b, a)	Report	Not coherent
Kattegatt/Skagerrak	2015	Inverts/Fish	Several	Larval disp	Moksnes et al. (2015)	Report	Not coherent
Kattegatt/Skagerrak	2016	Inverts/Fish	Several	Larval disp	Jonsson et al. (2016a, b)	Article	Not coherent

The MPA network has also been evaluated using set dispersal distances and benthic habitat maps. In the Baltic Sea this was done using set dispersal values of 25 and/or 50 km for a set of representative species, including bladderwrack *Fucus vesiculosus*, the red algae *Furcellaria lumbricalis*, Baltic tellin *Macoma baltica*, the isopod *Idotea baltica* and turbot *Psetta maxima* (Piekäinen and Korpinen 2007; HELCOM 2010, 2016; Wolters et al. 2015). The number of links (connectivity) between MPAs based on these distances and some species-specific values for the five selected species ranging from 1–100 km, depending on species, were examined. These studies also evaluated the other three coherence criteria by studying the size distribution of MPAs (*Adequacy*), the amount of habitats protected (*Representativity*) and how many areas of at least 24 ha of each habitat that were protected (*Replication*). In Kattegat-Skagerrak, one study was performed, where 50 and 80 km were used as set dispersal values for evaluating connectivity (Johnson et al. 2014) and the other three criteria were evaluated in a similar manner as those in the Baltic Sea. Results showed that the MPA network was only partly coherent (Table 3).

Using similar methods, but based on species-specific habitat requirements, species distributions and active migrations, Sundblad et al. (2011) evaluated the coherence of the Natura 2000 network in a 30 000-km^2^ archipelago in the Baltic Sea. Focus was to test connectivity and representativity, defined as amount of fish habitat protected by the network, for pike (*Esox lucius*), perch (*Perca fluviatilis*), pike-perch (*Sander lucioperca*) and roach (*Rutilus rutilus*). Both connectivity of all habitats and protected habitats were evaluated. Connectivity and representativity was found to be weak, and the map-based analyses identified areas in which the network could be strengthened. This method is particularly appropriate in complex coastal areas like archipelagos, lacking high resolution hydrodynamic maps, with patchy habitat distribution and where the dispersal is mainly through active migrations by individuals.

A study evaluating representativity of herring (*Clupea harengus*) spawning grounds within the Natura 2000 network along the Finnish coast was also performed by Bergström et al. (2007). Representativity was high in the Bothnian Bay where 40–50% of herring spawning grounds were protected, but coherence in terms of representativity was weak in the Gulf of Finland. Recently a more thorough evaluation was performed along the Finnish coast in which distribution maps for the most common species, key species and habitat forming species (e.g. pondweed *Potamogeton perfoliatus* bladderwrack *F. vesiculosus*, eelgrass *Z. marina* and blue mussels *Mytilus edulis*), threatened and red listed species were included (Virtanen et al. 2018). Maps of fish nursery grounds based on models by Kallasvuo et al. (2016) were also included. Only 27% of the most ecologically important areas were found to be protected by the existing MPA network. However, by expanding the network in appropriate areas, an expansion of as little as 1% of the network would double the coherence of ecologically important areas.

Besides uncertainties regarding species dispersal ranges, one main shortcoming in all analyses was that none but Virtanen et al. (2018) had taken threats and impacts into account. Virtanen et al. (2018) included threats like habitat loss, degradation and/or disturbance of habitats, in order to identify the ecologically most important (pristine) areas. All other analyses have assumed that habitats and organisms within MPAs are highly protected. In reality, protection may be rather low in many areas. Fishing regulations are for example rare. Fishing might, however, have negative effects on both fish abundances, bycatch species and benthic environments subjected to destructive fishing gears (Hammersland and Hjerpe Olausson 2011). Protected areas may also be subjected to pressure from coastal development, even in cases when conservation values are impacted. For example, Eriander et al. (2017) found that the existence of an MPA only marginally reduced the approval of applications for dock constructions in threatened eelgrass habitats on the Swedish west coast. This highlights the need to consider both threats, impacts and type of protection (nature reserve, national park, Natura 2000, HELCOM, OSPAR etc.) when evaluating the MPA network.

Outside of our study region, few studies testing the ecological coherence of MPA networks exist and most of them focus on connectivity and larval dispersal (e.g. Ross et al. 2017; Assis et al. 2021). A number of studies do, however, investigate the effect of connectivity on MPAs performance, mainly in tropical regions (e.g. Ortodossi et al. 2019; Steneck et al. 2009; Goetze et al. 2021) or incorporate connectivity in conservation prioritisation by identifying and studying connectivity between nursery and adult habitats in tropical seascapes (e.g. Weeks 2017). One major difference, and challenge, between our study area and other temperate or tropical seascapes are the strong environmental gradients resulting in a great mix of both marine and freshwater species with contrasting dispersal and migration strategies and patterns. Also, habitat patches are not always as distinct as e.g. coral reefs and seagrass beds in the tropics, where connectivity between juvenile and adult habitat can more easily be assessed. Recently a study by Rees et al. (2018) suggested ways to align the process of designating ecologically coherent MPA networks with economic development, environmental sustainability and social inclusion to achieve social-ecological coherence in MPA network design. This illustrates the need to also incorporate social aspects in MPA management and governance.

## Knowledge needs for improved connectivity analyses

Knowledge on species dispersal, both active and passive, in the Baltic Sea, Kattegat and Skagerrak is limited. Tagging studies have mainly been conducted among commercially important species in order to obtain distances of active migrations (Aro 2002; Saulamo and Neuman 2002; Drenner et al. 2012). Most tagging studies have been conducted on large fish, while information on migrations by invertebrates and small, non-commercial species of fish is scarce, although these species may be important for ecosystem functioning.

Information on passive dispersal and important areas to protect has mainly been obtained from modelling studies (Corell et al. 2012; Moksnes et al. 2015; Jonsson et al. 2016b, 2020). Modelling studies have also been done separately for herring, cod, sprat, European flounder and turbot in order to identify pathways of larval dispersal and nursery grounds (Florin et al. 2013; Hinrichsen et al. 2012a, b, 2017a, b).

The number of genetic studies reflecting dispersal or migration distances are also limited. Few studies presented distance estimate values and in many cases we had to extract the information from published maps. Genetic studies displaying genetic variation among populations gave an estimate of how far individuals may disperse. However, these studies focused on the individuals that disperse/migrate the furthest, since low levels of gene flow can even out genetic differences. Tagging studies that focus on dispersal distances performed by the majority of the population reflect scales relevant to population dynamics and may hence be more useful for evaluating ecological coherence of MPAs. Combining genetic studies with other methods, such as tagging and modelling, will provide a more comprehensive overview of connectivity. However, only very few studies exist for the Baltic Sea, i.e. De Wit et al. (2020) combining genetics with a biophysical model of connectivity for the isopod *Idotea balthica*, Östergren et al. (2012) combining genetics with acoustic tagging to follow spawning migrations of sea trout and Larsson et al. (2015) combining genetics with otolith chemistry to track pike migration in the Baltic Sea. In Kattegat-Skagerrak, a limited amount of studies have combined genetics with larval dispersal models in cod (Jonsson et al. 2016a; Barth et al. 2017) and eelgrass (Jahnke et al. 2018). Jahnke et al. (2020) further combined genetics with dispersal models to identify sites for seagrass conservation in the same region.

Genetic methods can be used to directly estimate migration for species not affected by stocking. Using genetic markers with high coverage, the source population for individuals can be identified with high certainty. SNPs (Single Nucleotide Polymorphisms) associated with genes under selection has been used for stock identification in commercially fished species. For example, an individual cod can be assigned to the western or eastern Baltic Sea stock with high certainty (Nielsen et al. 2012). However, to date, genetic methods have only been used to study connectivity among MPAs or between MPAs and non-protected areas in a few cases worldwide, even though it is a promising tool (Jenkins and Stevens 2018). By identifying the origin of single individuals, migrants can be identified. It is also possible to use parentage analysis to identify where an individual’s parents come from in order to determine if migrants also reproduce in new environments. For example in Swedish coastal and marine waters, the reproductive success of wild versus farmed salmon was studied (Dannewitz et al. 2004). However, using genetics to study connectivity directly requires rigorous sampling. If migration is low among populations or MPAs, many individuals need to be sampled in order to catch potential migrants. Furthermore, parentage analysis requires comprehensive sampling of the parent population. Nevertheless, there are also advantages to using genetic methods to study connectivity. Genetic markers are present within each individual and can be used as individual tags, without the requirement to undertake large tagging studies.

With information on relevant migration and dispersal distances, including both passive and active movement, modelling studies can be used to identify; (1) how large MPAs need to be in order to obtain viable populations, (2) important recruitment areas contributing to connectivity (connectivity hotspots), (3) optimum networks of MPAs, (4) optimal areas in which to extend the MPA network and (5) dispersal barriers to consider in MPA design. Evaluations of MPA networks in the Baltic Sea and Kattegat-Skagerrak have so far focused on larval dispersal. To evaluate the connectivity of MPA networks for species that disperse mainly through active movement, a distance-based method including active migration distances may be used. This primarily includes many coastal species with freshwater origin in the Baltic Sea, but also some marine fish and invertebrates. Such analyses have been done for pike, perch, pike-perch and roach in parts of the Baltic Sea (Sundblad et al. 2011) but could be applied to a larger area and more species when habitat maps become available. The distance-based method is appropriate for testing the coherence of MPAs in complex coastal environments with patchy habitat distribution and where the majority of species disperse by active migrations rather than passive dispersal. Habitat maps on migration corridors would also be very useful (Krost et al. 2018). However, maps and studies like these are lacking for both the Baltic Sea and Kattegat-Skagerrak.

In conclusion, there is a growing need to study dispersal dynamics among species, particularly invertebrates and non-commercial fishes. Studies combining various methods (tagging, otolith chemistry, genetics and modelling) are encouraged to gain a broader knowledge on both home ranges affecting population dynamics and maximum distances affecting genetic variation in populations. There is also a demand for comprehensive species distribution maps that can be used to perform spatial analyses on connectivity with both larval dispersal models and analyses of active migrations. Adequate habitat maps can be produced by species distribution modelling based on a thorough empirical sampling program, similar to in Virtanen et al. (2018). Large-scale studies on larval dispersal have recently been performed in both the Baltic Sea and Kattegat-Skagerrak (Berglund et al. 2012; Corell et al. 2012; Moksnes et al. 2014b, 2016, Jonsson et al. 2016b; Hinrichsen et al. 2017a; Jonsson et al. 2020). In the future, evaluations including higher resolution hydrodynamics can be performed, enabling analysis of larval dispersal also in topographically complex coastal areas. Similarly, there is a need to perform comprehensive analyses on coastal species with short larval dispersal performing active migrations. This is true for both the Baltic Sea and Kattegat-Skagerrak. Furthermore, threats to ecological connectivity, including climate change and other human pressures like coastal development, that potentially limit dispersal between MPAs and lowers the resilience to environmental change, should be incorporated. These analyses can be used to identify areas of special importance for connectivity and to evaluate their sensitivity to different pressures, providing information for marine spatial planning, green infrastructure and habitat restoration.

## Supplementary Information

Below is the link to the electronic supplementary material.

Supplementary file1 (PDF 1085 kb)
