# Supplementary material for: Ecological connectivity of the marine protected area network in the Baltic Sea, Kattegat and Skagerrak: Current knowledge and management needs
Source: Ambio. 2021 Dec 29;51(6):1485–503. doi: 10.1007/s13280-021-01684-x (PMC9005595; doi:10.1007/s13280-021-01684-x)
Supplement: Supplementary file 1 — Supplementary file1 (PDF 1085 kb) [file 13280_2021_1684_MOESM1_ESM.pdf]

Title Page

Title

Ecological connectivity of the marine protected area network in the Baltic Sea, Kattegat and Skagerrak: current knowledge and management needs

Authors: Charlotte Berkström, Lovisa Wennerström, and Ulf Bergström

Word count

12 283

Acknowledgements

We wish to thank the Swedish Agency for Marine and Water Management (SwAM) for funding this project and Leonard Sandin for valuable comments on an earlier version of the manuscript. We also wish to thank Mårten Erlandsson for help with images and two anonymous reviewers for helpful comments.

Author Contact details

Charlotte Berkström
Address: Department of Aquatic Resources, Swedish University of Agricultural Sciences, Institute of Coastal Research, Skolgatan 6, SE 742 42 Öregrund, Sweden
e-mail: charlotte.berkstrom@slu.se
Phone: + 46 10 4784165

Lovisa Wennerström
Address: Department of Aquatic Resources, Swedish University of Agricultural Sciences, Institute of Coastal Research, Skolgatan 6, SE 742 42 Öregrund, Sweden
e-mail: lovisa.wennerstrom@slu.se

Ulf Bergström
Address: Department of Aquatic Resources, Swedish University of Agricultural Sciences, Institute of Coastal Research, Skolgatan 6, SE 742 42 Öregrund, Sweden
e-mail: ulf.bergstrom@slu.se

Title: Connectivity of the marine protected area network in the Baltic Sea, Skagerrak and Kattegat: current knowledge and management needs

Appendix S1

Search strings in Web of Science: (nov 2020)

Connectivity* coherence* marine protected area*
Connectivity* coherence* marine reserve*
Connectivity* coherence* marine spatial planning*
Connectivity* coherence* Baltic Sea*
Connectivity* coherence* Swedish west coast*
Connectivity* coherence* Skagerrak*
Connectivity* coherence* Kattegat*
Ecological coherence* marine protected area*
Ecological coherence* marine reserve*
Ecological coherence* marine spatial planning*
Ecological coherence* Baltic Sea*
Ecological coherence* Swedish west coast*
Ecological coherence* Skagerrak*
Ecological coherence* Kattegat*
Connectivity* Baltic Sea*
Connectivity* Swedish west coast*
Connectivity* Skagerrak*
Connectivity* Kattegat*
Dispersal* Baltic Sea*
Dispersal* Swedish west coast*
Dispersal* Skagerrak*
Dispersal* Kattegat*
Fish migration* Baltic Sea*
Fish migration* Swedish west coast*
Fish migration* Skagerrak*
Fish migration* Kattegat*
Home range* fish* Baltic Sea*
Home range* fish* Swedish west coast*
Home range* fish* Skagerrak*
Home range* fish* Kattegat*
Tagging* Baltic Sea*
Tagging* Swedish west coast*
Tagging* Skagerrak*
Tagging* Kattegat*
Nursery* Baltic Sea*
Nursery* Swedish west coast*
Nursery* Skagerrak*
Nursery* Kattegat*
Spawning migration* Baltic Sea*
Spawning migration* Swedish west coast*
Spawning migration* Skagerrak*
Spawning migration* Kattegat*
Fish* Swedish west coast*
Marine green infrastructure*
green infrastructure* Connectivity* aquatic*
green-blue infrastructure*

green infrastructure* connectivity* Europe*
ecological corridor* Baltic Sea*
ecological corridor * Skagerrak*
ecological corridor * Kattegat*

Table S1. References to all distance values per species in table 2. All references are from the Baltic Sea, Skagerrak and/or Kattegat unless stated otherwise.

Species	Common name	References on dispersal and movement measurements
Abramis brama	Freshwater bream	Aro (1989)
Acipenser oxyrinchus	Atlantic Sturgeon	Stakėnas & Pilinkovskij (2019)
Ammodytes marinus	Lesser sand-eel	Jiménez-Mena et al. (2020)
Amphibalanus improvisus	Bay barnacle	Corell et al. (2012)
Anguilla anguilla	European eel	Dannewitz et al. (2005), Bergström et al. (2007), Enbody et al. (2021) Fishbase.com
Ascidia mentula	Tunicate	Havenhand (1991)
Ascophyllum nodosum	Knotted wrack	Dudgeon et al. (2001) - from USA, Coyer et al. (2003) and references in paper
Aurelia aurita	Moon jellyfish	Moksnes et al. (2014b)
Cancer pagurus	Edible/Brown crab	Karlsson & Christiansen (1996) and references in paper – from Norway, Ungfors et al. (2007), Ungfors et al. (2009) and references in paper, Artfakta.se
Carcinus maenas	Eruopean shore crab	Moksnes et al. (2014a)
Ciona intestinalis	Vase tunicate	Hudson et al. (2020)
Clupea harengus	Atlantic herring	Weber (1975), Saulamo & Neuman (2002), Bekkevold et al. (2005), Jörgensen et al. (2005), Bergström et al. (2007), Gaggiotti et al. (2009), Larsson et al. (2010), Bekkevold et al. (2011), Corander et al. (2013), Teacher et al. (2013), Claussen et al. (2015) and references in paper, Bekkevold et al. (2016)
Coregonus maraena	Maraena whitefish	Lehtonen & Himberg (1992), Bergström et al. (2007), Olsson et al. (2012)
Coregonus maraena	Maraena whitefish	Aro (1989), Lehtonen & Himberg (1992), Bergström et al. (2007), Fishbase.com
Coryphaenoides rupestris	Roundnose grenadier	Delaval et al. (2018)
Cottus gobio	Bullhead	Corell et al. (2012)
Ctenolabrus rupestris	Goldsinny wrasse	Sayer (2001) – from UK
Cyclopterus lumpus	Lumpfish	Whittaker et al. (2018)
Esox lucius	Pike	Aro (1989), Karås & Lehtonen (1993), Laikre et al. (2005), Bergström et al. (2007), Bekkevold et al. (2015), Larsson et al. (2015), Tibblin et al. (2016), Wennerström et al. (2016), Jacobsen et al. (2017), Nordahl et al. (2019), Möller et al. (2021), Fishbase.com
Fucus radicans	Baltic bladderwrack	Coyer et al. (2003) and references in paper, Jonsson et al. (2018) and references in paper
Fucus serratus	Toothed/Serrated wrack	Coyer et al. (2003) and references in paper
Fucus vesiculosus	Bladderwrack	Coyer et al. (2003) and references in paper, Tatarenkov et al. (2007), Pereyra et al. (2013), Rothäusler et al. (2015), Jonsson et al. (2018) and references in paper

Gadus morhua	Atlantic cod	Baltic Sea: Saulamo & Neuman (2002), Nielsen et al. (2003), Nielsen et al. (2005), Bergström et al. (2007), Kijewska et al. (2011), Stroganov et al. (2013); Kattegatt/Skagerrak: Knutsen et al. (2003), Jorde et al. (2007), Øresland & André (2008), Knutsen et al. (2011), André et al. (2016), Barth et al. (2017), Spies et al. (2018), Svedäng et al. (2018), Fishbase.com
Gasterosteus aculeatus	Three-spined stickleback	DeFaveri et al. (2012), DeFaveri et al. (2013b), DeFaveri et al. (2013a), Guo et al. (2015)
Gobius niger	Black goby	Corell et al. (2012)
Gymnocephalus cernuus	Ruffe	Gutsch & Hoffman (2016)
Hediste diversicolor	Ragworm	Einfeldt et al. (2014)
Homarus gammarus	European Lobster	Moland et al. (2011b), Moland et al. (2011a), Huserbråten et al. (2013), Moland et al. (2013b), Moland et al. (2013a), Øresland & Ulmestrand (2013), Thorbjörnsen et al. (2018)
Idotea balthica	Baltic isopod	De Wit et al. (2020)
Labrus bergylta	Ballan wrasse	Villegas-Ríos (2013) - from Spain
Leuciscus idus	Ide	Rohtla et al. (2015)
Liparis liparis	Striped seasnail	Corell et al. (2012)
Littorina fabalis	Flat periwinkle	Erlandsson et al. (1998)
Littorina littorea	Common periwinkle	Johannesson et al. (1988)
Littorina saxatilis	Rough periwinkle	Janson et al. (1983), Johannesson & Johannesson (1995), Erlandsson et al. (1998), Panova et al. (2006)
Lophelia pertusa	Spider hazards	Dahl et al. (2012), Moksnes et al. (2014b)
Lota lota	Burbot	Aro (1989)
Merluccius merluccius	European hake	Westgaard et al. (2017)
Modiolus modiolus	Northern horsemussel	Johnson et al. (2008), www.marlin.ac.uk - From UK
Mytilus edulis	Blue mussel	Gilg et al. (2003), Corell et al. (2012), Larsson et al. (2016), Strelkov et al. (2017), Stuckas et al. (2017)
Nerophis ophidion	Straightnose pipefish	Corell et al. (2012)
Ostrea edulis	Flat oyster	Kinlan och Gaines (2003), Johnson et al. (2008)
Perca fluviatilis	European Perch	Karås (1996), Järv (2000), Bergström et al. (2007), Bergek & Björklund (2009), Olsson et al. (2011), Ahlbeck Bergendahl et al. (2017), Hansson et al. (2019), Artfakta.se
Pholis gunnellus	Rock gunnel	Corell et al. (2012)
Platichthys flesus	European flounder	Florin (2005), Hemmer-Hansen et al. (2007b), Hemmer-Hansen et al. (2007a), Florin & Höglund (2008), ICES (2010) and references in paper, Florin et al. (2013), Momigliano et al. (2017), Momigliano et al. (2018), Reis-Santos et al. (2018)
Platichthys solemdali	European flounder	Momigliano et al. (2017), Momigliano et al. (2018)

Pleuronectes platessa	European plaice	Aro (1989), Ulrich et al. (2017)
Pomatoschistus minutus	Sand goby	Larmuseau et al. (2010a), Larmuseau et al. (2010b), Corell et al. (2012),
Pygospio elegans	Polychaete	Corell et al. (2012)
Ruppia maritima	Beaked tasselweed	Triest & Sierens (2015), Triest et al. (2018)
Ruppia spiralis	Widgeongrass	Triest et al. (2018)
Salmo salar	Atlantic salmon	Jutila et al. (2003), Bergström et al. (2007), Palm et al. (2008), Pedersen et al. (2007), Tonteri et al. (2007), Palmé et al. (2012), Whitlock et al. (2018), Miettinen et al. (2021)
Rutilus rutilus	Roach	Hansson et al. (2019)
Salmo trutta	Sea trout	Kalio-Nyberg et al. (2002), Bergström et al. (2007), Degerman et al. (2012), Östergren et al. (2012), Östergren & Nilsson (2012), Jonsson & Jonsson (2014), Kalio-Nyberg et al. (2017)
Sander lucioperca	Pike-perch	Saulamo & Thoresson (2005), Bergström et al. (2007), Björklund et al. (2007), Säisä et al. (2010), Andersson et al. (2015), Ložys et al. (2017)
Scomber scombrus	Atlantic mackerel	Jansen et al. (2012)
Scophthalmus maximus	Turbot	Nielsen et al. (2004), Florin (2005), Bergström et al. (2007), ICES (2010) and references in paper, Florin & Höglund (2007), Florin et al. (2013)
Skeletonema marioni	Diatom	Sefbom et al. (2018)
Solea solea	Common sole	Symonds & Rogers (1995) – from UK, Cuveliers et al. (2012)
Sprattus sprattus	European sprat	Limborg et al. (2009)
Symphodus melops	Corkwing wrasse	Halvorsen et al. (2016), Faust et al. (2018), Mattingsdal et al. (2020)
Zoarces viviparus	Eelpout	Bergek et al. (2012), Kinitz et al. (2013)
Zostera marina	Eelgrass	Orth et al. (1994) - from USA, Reusch (2002), Källström et al. (2008), Jahnke et al. (2018) and references in paper, Triest et al. (2018) and references in paper
Zostera noltii	Dwarf eelgrass	Chust et al. (2013) - from Spain

References

Ahlbeck Bergendahl, I., P. B. Holliland, S. Hansson, and O. Karlöf. 2017. Feeding range of age 1+ year Eurasian perch *Perca fluviatilis* in the Baltic Sea. Journal of Fish Biology 90:2060-2072.

Andersson, M., E. Degerman, J. Persson, and H. Ragnarsson-Stabo. 2015. Movements, recapture rate and length increment of tagged pikeperch (*Sander lucioperca*) – a basis for management in large lakes. Fisheries Management and Ecology 22:450-457.

André, C., H. Svedäng, H. Knutsen, G. Dahle, P. Jonsson, A.-K. Ring, M. Sköld, and P. E. Jorde. 2016. Population structure in Atlantic cod in the eastern North Sea-Skagerrak-Kattegat: early life stage dispersal and adult migration. BMC Research Notes 9:63.

Aro, E. 1989. A review of fish migration patterns in the Baltic. Rapp. P.-v. Réun. Cons. int. Explor. Mer:72-96.
Barth, J. M. I., P. R. Berg, P. R. Jonsson, S. Bonanomi, H. Corell, J. Hemmer-Hansen, K. S. Jakobsen, K. Johannesson, P. E. Jorde, H. Knutsen, P.-O. Moksnes, B. Star, N. C. Stenseth, H. Svedäng, S. Jentoft, and C. André. 2017. Genome architecture enables local adaptation of Atlantic cod despite high connectivity. Molecular Ecology 26:4452-4466.
Bekkevold, D., C. Aandré, T. G. Dahlgren, L. A. W. Clausen, E. Torstensen, H. Mosegaard, G. R. Carvalho, T. B. Christensen, E. Norlinder, and D. E. Ruzzante. 2005. Environmental correlates of population differentiation in Atlantic herring. Evolution 59:2656-2668.
Bekkevold, D., L. A. W. Clausen, S. Mariani, C. André, E. M. C. Hatfield, E. Torstensen, N. Ryman, G. R. Carvalho, and D. E. Ruzzante. 2011. Genetic mixed-stock analysis of Atlantic herring populations in a mixed feeding area. Marine Ecology Progress Series 442:187-199.
Bekkevold, D., R. Gross, T. Arula, S. J. Helyar, and H. Ojaveer. 2016. Outlier loci detect intraspecific biodiversity amongst spring and autumn spawning herring across local scales. PLoS ONE 11:e0148499.
Bekkevold, D., L. Jacobsen, J. Hemmer-Hansen, S. Berg, and C. Skov. 2015. From regionally predictable to locally complex population structure in a freshwater top predator: river systems are not always the unit of connectivity in Northern Pike *Esox lucius*. Ecology of Freshwater Fish 24:305-316.
Bergek, S., and M. Björklund. 2009. Genetic and morphological divergence reveals local subdivision of perch (*Perca fluviatilis* L.). Biological Journal of the Linnean Society 96:746-758.
Bergek, S., F. Franzén, M. Quack, A. Hochkirch, T. Kinitz, T. Prestegaard, and M. Appelberg. 2012. Panmixia in *Zoarces viviparus*: implications for environmental monitoring studies. Journal of Fish Biology 80:2302-2316.
Bergström, L., S. Korpinen, U. Bergström, and Å. Andersson. 2007. Essential fish habitats and fish migration patterns in the Northern Baltic Sea.
Björklund, M., T. Aho, and L. C. Larsson. 2007. Genetic differentiation in pikeperch (*Sander lucioperca*): the relative importance of gene flow, drift and common history. Journal of Fish Biology 71:264-278.
Chust, G., A. Albaina, A. Aranburu, Á. Borja, O. E. Diekmann, A. Estonba, J. Franco, J. M. Garmendia, M. Iriondo, I. Muxika, F. Rendo, J. G. Rodríguez, O. Ruiz-Larrañaga, E. A. Serrão, and M. Valle. 2013. Connectivity, neutral theories and the assessment of species vulnerability to global change in temperate estuaries. Estuarine, Coastal and Shelf Science 131:52-63.
Clausen, L. A. W., K.-J. Stæhr, A. Rindorf, and H. Mosegaard. 2015. Effect of spatial differences in growth on distribution of seasonally co-occurring herring *Clupea harengus* stocks. Journal of Fish Biology 86:228-247.
Corander, J., K. K. Majander, L. Cheng, and J. Merilä. 2013. High degree of cryptic population differentiation in the Baltic Sea herring *Clupea harengus*. Molecular Ecology 22:2931-2940.
Corell, H., P. O. Moksnes, A. Engqvist, K. Döös, and P. R. Jonsson. 2012. Depth distribution of larvae critically affects their dispersal and the efficiency of marine protected areas. Marine Ecology Progress Series 467:29-46.
Coyer, J. A., A. F. Peters, W. T. Stam, and J. L. Olsen. 2003. Post-ice age recolonization and differentiation of *Fucus serratus* L. (Phaeophyceae; Fucaceae) populations in Northern Europe. Molecular Ecology 12:1817-1829.
Cuveliers, E. L., M. H. D. Larmuseau, B. Hellemans, S. L. N. A. Verherstraeten, F. A. M. Volckaert, and G. E. Maes. 2012. Multi-marker estimate of genetic connectivity of sole (*Solea solea*) in the North-East Atlantic Ocean. Marine Biology 159:1239-1253.
Dahl, M. P., R. T. Pereyra, T. Lundälv, and C. André. 2012. Fine-scale spatial genetic structure and clonal distribution of the cold-water coral *Lophelia pertusa*. Coral Reefs 31:1135-1148.

Dannewitz, J., G. E. Maes, L. Johansson, H. Wickström, F. A. M. Volckaert, and T. Järvi. 2005. Panmixia in the European eel: a matter of time.. Proceedings of the Royal Society B: Biological Sciences 272:1129-1137.
De Wit, P., P. R. Jonsson, R. T. Pereyra, M. Panova, C. André, and K. Johannesson. 2020. Spatial genetic structure in a crustacean herbivore highlights the need for local considerations in Baltic Sea biodiversity management. Evolutionary Applications 13:974-990.
DeFaveri, J., P. R. Jonsson, and J. Merilä. 2013a. Heterogeneous genomic differentiation in marine threespine sticklebacks: adaptation along an environmental gradient. Evolution 67:2530-2546.
DeFaveri, J., T. Shikano, N. I. A. Ghani, and J. Merilä. 2012. Contrasting population structures in two sympatric fishes in the Baltic Sea basin. Marine Biology 159:1659-1672.
DeFaveri, J., T. Shikano, Y. Shimada, and J. Merilä. 2013b. High degree of genetic differentiation in marine three-spined sticklebacks (*Gasterosteus aculeatus*). Molecular Ecology 22:4811-4828.
Degerman, E., K. Leonardsson, and H. Lundqvist. 2012. Coastal migrations, temporary use of neighbouring rivers, and growth of sea trout (*Salmo trutta*) from nine northern Baltic Sea rivers. ICES Journal of Marine Science 69:971-980.
Delaval, A., G. Dahle, H. Knutsen, J. Devine, and A. G. V. Salvanes. 2018. Norwegian fjords contain sub-populations of roundnose grenadier *Coryphaenoides rupestris*, a deep-water fish. Marine Ecology Progress Series 586:181-192.
Dudgeon, S., J. E. Kübler, W. A. Wright, R. L. Vadas Sr, and P. S. Petraitis. 2001. Natural variability in zygote dispersal of *Ascophyllum nodosum* at small spatial scales. Functional Ecology 15:595-604.
Einfeldt, A. L., J. R. Doucet, and J. A. Addison. 2014. Phylogeography and cryptic introduction of the ragworm *Hediste diversicolor* (Annelida, Nereididae) in the Northwest Atlantic. Invertebrate Biology 133:232-241.
Enbody, E. D., M. E. Pettersson, C. G. Sprehn, S. Palm, H. Wickström, and L. Andersson. 2021. Ecological adaptation in European eels is based on phenotypic plasticity. Proceedings of the National Academy of Sciences 118:e2022620118.
Erlandsson, J., E. Rolán-Alvarez, and K. Johannesson. 1998. Migratory differences between ecotypes of the snail *Littorina saxatilis* on Galician rocky shores. Evolutionary Ecology 12:913-924.
Faust, E., T. Halvorsen Kim, P. Andersen, H. Knutsen, and C. André. 2018. Cleaner fish escape salmon farms and hybridize with local wrasse populations. Royal Society Open Science 5:171752.
Florin, A.-B., and J. Höglund. 2007. Absence of population structure of turbot (*Psetta maxima*) in the Baltic Sea. Molecular Ecology 16:115-126.
Florin, A. B. 2005. Flatfishes in the Baltic Sea - a review of biology and fishery with a focus on Swedish conditions.
Florin, A. B., U. Bergström, D. Ustups, K. Lundström, and P. R. Jonsson. 2013. Effects of a large northern European no-take zone on flatfish populations. Journal of Fish Biology 83:939-962.
Florin, A. B., and J. Höglund. 2008. Population structure of flounder (*Platichthys flesus*) in the Baltic Sea: differences among demersal and pelagic spawners. Heredity 101:27-38.
Gaggiotti, O. E., D. Bekkevold, H. B. H. Jørgensen, M. Foll, G. R. Carvalho, C. Andre, and D. E. Ruzzante. 2009. Disentangling the effects of evolutionary, demographic and environmental factors influencing genetic structure of natural populations: Atlantic herring as a case study. Evolution 63:2939-2951.
Gilg, M. R., and T. J. Hilbish. 2003. The geography of marine larval dispersal: coupling genetics with fine-scale physical oceanography. Ecology 84:2989-2998.

Guo, B., J. DeFaveri, G. Sotelo, A. Nair, and J. Merilä. 2015. Population genomic evidence for adaptive differentiation in Baltic Sea three-spined sticklebacks. BMC Biology 13:19.

Gutsch, M., and J. Hoffman. 2016. A review of Ruffe (*Gymnocephalus cernua*) life history in its native versus non-native range. Reviews in Fish Biology and Fisheries 26:213-233.

Halvorsen, K. T., T. K. Sørdalen, L. A. Vøllestad, A. B. Skiftesvik, S. H. Espeland, and E. M. Olsen. 2016. Sex- and size-selective harvesting of corkwing wrasse (*Symphodus melops*)—a cleaner fish used in salmonid aquaculture. ICES Journal of Marine Science 74:660-669.

Hansson, S., U. Larsson, and J. Walve. 2019. Site fidelity in perch (*Perca fluviatilis*) and roach (*Rutilus rutilus*) in two Baltic Sea coastal areas. Estuarine, Coastal and Shelf Science 226:106261.

Havenhand, J. N. 1991. Fertilisation and the potential for dispersal of gametes and larvae in the Solitary Ascidian *Ascidia Mentula* Müller. Ophelia 33:01-15.

Hemmer-Hansen, J., E. E. Nielsen, J. Frydenberg, and V. Loeschcke. 2007a. Adaptive divergence in a high gene flow environment: Hsc70 variation in the European flounder (*Platichthys flesus* L.). Heredity 99:592-600.

Hemmer-Hansen, J., E. E. Nielsen, P. Gronkjaer, and V. Loeschcke. 2007b. Evolutionary mechanisms shaping the genetic population structure of marine fishes; lessons from the European flounder (*Platichthys flesus* L.). Molecular Ecology 16:3104-3118.

Hudson, J., K. Johannesson, C. D. McQuaid, and M. Rius. 2020. Secondary contacts and genetic admixture shape colonization by an amphiatlantic epibenthic invertebrate. Evolutionary Applications 13:600-612.

Huserbråten, M. B. O., E. Moland, H. Knutsen, E. M. Olsen, C. André, and N. C. Stenseth. 2013. Conservation, spillover and gene flow within a network of Northern European marine protected areas. PLoS ONE 8:e73388.

ICES. 2010. Report of the ICES/HELCOM Workshop on Flatfish in the Baltic Sea (WKFLABA), 8 - 11 November 2010, Öregrund, Sweden. ICES CM 2010/ACOM:68. 85pp.

Jacobsen, L., D. Bekkevold, S. Berg, N. Jepsen, A. Koed, K. Aarestrup, H. Baktoft, and C. Skov. 2017. Pike (*Esox lucius* L.) on the edge: consistent individual movement patterns in transitional waters of the western Baltic. Hydrobiologia 784:143-154.

Jahnke, M., P. R. Jonsson, P. O. Moksnes, L. O. Loo, J. M. Nilsson , and J. L. Olsen. 2018. Seascape genetics and biophysical connectivity modelling support conservation of the seagrass *Zostera marina* in the Skagerrak–Kattegat region of the eastern North Sea. Evolutionary Applications 11:645-661.

Jansen, T., A. Campbell, C. Kelly, H. Hátún, and M. R. Payne. 2012. Migration and fisheries of north east Atlantic mackerel (*Scomber scombrus*) in autumn and winter. PLoS ONE 7:e51541.

Janson, K. 1983. Selection and migration in two distinct phenotypes of Littorina saxatilis in Sweden. Oecologia 59:58-61.

Jiménez-Mena, B., A. Le Moan, A. Christensen, M. van Deurs, H. Mosegaard, J. Hemmer-Hansen, and D. Bekkevold. 2020. Weak genetic structure despite strong genomic signal in lesser sandeel in the North Sea. Evolutionary Applications 13:376-387.

Johannesson, K. 1988. The paradox of Rockall: why is a brooding gastropod (*Littorina saxatilis*) more widespread than one having a planktonic larval dispersal stage (*L. littorea*)? Marine Biology 99:507-513.

Johannesson, K., and B. Johannesson. 1995. Dispersal and population expansion in a direct developing marine snail (*Littorina saxatilis*) following a severe population bottleneck. Hydrobiologia 309:173-180.

Johnson, M. P., T. P. Crowe, R. McAllen, and A. L. Allcock. 2008. Characterizing the marine Natura 2000 network for the Atlantic region. Aquatic Conservation: Marine and Freshwater Ecosystems 18:86-97.

Jonsson, B., and N. Jonsson. 2014. Naturally and hatchery produced European trout *Salmo trutta*: do their marine survival and dispersal differ? Journal of Coastal Conservation 18:79-87.

Jonsson, P. R., J. Kotta, H. C. Andersson, K. Herkül, E. Virtanen, A. N. Sandman, and K. Johannesson. 2018. High climate velocity and population fragmentation may constrain climate-driven range shift of the key habitat former *Fucus vesiculosus*. Diversity and Distributions 24:892-905.

Jorde, P. E., H. Knutsen, S. H. Espeland, and N. C. Stenseth. 2007. Spatial scale of genetic structuring in coastal cod Gadus morhua and geographic extent of local populations. Marine Ecology Progress Series 343:229-237.

Jutila, E., E. Jokikokko, I. Kallio-Nyberg, I. Saloniemi, and P. Pasanen. 2003. Differences in sea migration between wild and reared Atlantic salmon (*Salmo salar* L.) in the Baltic Sea. Fisheries Research 60:333-343.

Järv, L. 2000. Migrations of the perch (Perca fluviatilis L.) in the coastal waters of Western Estonia. Proc. Estonian Acad. Sci. Biol. Ecol. 49:270-276.

Jörgensen, H. B. H., M. M. Hansen, D. Bekkevold, D. E. Ruzzante, and V. Loeschcke. 2005. Marine landscapes and population genetic structure of herring (*Clupea harengus* L.) in the Baltic Sea. Molecular Ecology 14:3219-3234.

Kallio-Nyberg, I., A. Saura, and P. Ahlfors. 2002. Sea migration pattern of two sea trout (*Salmo trutta*) stocks released into the Gulf of Finland. Ann. Zool. Fennici 39:221-235.

Kallio-Nyberg, I., L. Veneranta, I. Saloniemi, E. Jutila, and T. Pakarinen. 2017. Spatial distribution of migratory Salmo trutta in the northern Baltic Sea. Boreal Environmental Research 22:431-444.

Karlsson, K., and M. F. Christiansen. 1996. Occurrence and population composition of the edible crab (C*ancer pagurus*) on rocky shores of an islet on the South Coast of Norway. Sarsia 81:307-314.

Karås, P. 1996. Basic abiotic conditions for production of perch (*Perca fluviatilis* L.) young-of-the-year in the Gulf of Bothnia. Annales Zoologici Fennici 33:371-381.

Karås, P., and H. Lehtonen. 1993. Patterns of movement and migration in pike (*Exos lucius* L.) in the Baltic Sea. Nordic Journal of Freshwater Research:72-79.

Kijewska, A., B. Wiecaszek, and T. Kijewski. 2011. Analysis of population and taxonomical structure of Atlantic cod Gadus horhua (Actinopterygii: Gadiformes: Gadidae) from the Baltic Sea with use of microsatellite DNA. Acta Ichthyologica Et Piscatoria 41:307-314.

Kinitz, T., M. Quack, M. Paulus, M. Veith, S. Bergek, J. Strand, A. Tuvikene, A. Soirinsuo, and A. Hochkirch. 2013. Strong isolation-by-distance in the absence of genetic population structure in the eelpout (Zoarces viviparus, Linnaeus 1758). Ecological Indicators 27:116-122.

Kinlan, B. P., and S. D. Gaines. 2003. Propagule dispersal in marine and terrestrial environments: a community perspective. Ecology 84:2007-2020.

Knutsen, H., P. E. Jorde, C. André, and N. C. Stenseth. 2003. Fine-scaled geographical population structuring in a highly mobile marine species: the Atlantic cod. Molecular Ecology 12:385-394.

Knutsen, H., E. M. Olsen, P. E. Jorde, S. H. Espeland, C. André, and N. C. Stenseth. 2011. Are low but statistically significant levels of genetic differentiation in marine fishes 'biologically meaningful'? A case study of coastal Atlantic cod. Molecular Ecology 20:768-783.

Källström, B., A. Nyqvist, P. Åberg, M. Bodin, and C. André. 2008. Seed rafting as a dispersal strategy for eelgrass (*Zostera marina*). Aquatic Botany 88:148-153.

Laikre, L., L. M. Miller, A. Palmé, S. Palm, A. R. Kapuscinski, G. Thoresson, and N. Ryman. 2005. Spatial genetic structure of northern pike (*Esox lucius*) in the Baltic Sea. Molecular Ecology 14:1955-1964.

Larmuseau, M. H. D., J. A. M. Raeymaekers, B. Hellemans, J. K. J. Van Houdt, and F. A. M. Volckaert. 2010a. Mito-nuclear discordance in the degree of population differentiation in a marine goby. Heredity 105:532.

Larmuseau, M. H. D., K. Vancampenhout, J. A. M. Raeymaekers, J. K. J. Van Houdt, and F. A. M. Volckaert. 2010b. Differential modes of selection on the rhodopsin gene in coastal Baltic and North Sea populations of the sand goby, *Pomatoschistus minutus*. Molecular Ecology 19:2256-2268.

Larsson, A. I., L. M. Granhag, and P. R. Jonsson. 2016. Instantaneous flow structures and opportunities for larval settlement: barnacle larvae swim to settle. PLoS ONE 11:e0158957.

Larsson, L. C., L. Laikre, C. André, T. G. Dahlgren, and N. Ryman. 2010. Temporally stable genetic structure of heavily exploited Atlantic herring (*Clupea harengus*) in Swedish waters. Heredity 104:40.

Larsson, P., P. Tibblin, P. Koch-Schmidt, O. Engstedt, J. Nilsson, O. Nordahl, and A. Forsman. 2015. Ecology, evolution, and management strategies of northern pike populations in the Baltic Sea. Ambio 44:451-461.

Lehtonen, H., and M. Himberg. 1992. Baltic Sea migration patterns of anadromous, Coregonus Lavaretus (L) S. Str., and sea-spawning European whitefish, C.L. Widegreni Malmgren. Pol. Arch. Hydrobiol. 39:463-472.

Limborg, M. T., J. S. Pedersen, J. Hemmer-Hansen, J. Tomkiewicz, and D. Bekkevold. 2009. Genetic population structure of European sprat *Sprattus sprattus*: differentiation across a steep environmental gradient in a small pelagic fish. Marine Ecology Progress Series 379:213-224.

Ložys, L., J.-C. Shiao, Y. Iizuka, A. Minde, Ž. Pūtys, E. Jakubavičiūtė, J. Dainys, H. Gorfine, and W.-N. Tzeng. 2017. Habitat use and migratory behaviour of pikeperch *Sander lucioperca* in Lithuanian and Latvian waters as inferred from otolith Sr:Ca ratios. Estuarine, Coastal and Shelf Science 198:43-52.

Mattingsdal, M., P. E. Jorde, H. Knutsen, S. Jentoft, N. C. Stenseth, M. Sodeland, J. I. Robalo, M. M. Hansen, C. André, and E. Blanco Gonzalez. 2020. Demographic history has shaped the strongly differentiated corkwing wrasse populations in Northern Europe. Molecular Ecology 29:160-171.

Miettinen, A., S. Palm, J. Dannewitz, E. Lind, C. R. Primmer, A. Romakkaniemi, J. Östergren, and V. L. Pritchard. 2021. A large wild salmon stock shows genetic and life history differentiation within, but not between, rivers. Conservation Genetics 22:35-51.

Moksnes, P.-O., H. Corell, K. Tryman, R. Hordoir, and P. R. Jonsson. 2014a. Larval behavior and dispersal mechanisms in shore crab larvae (*Carcinus maenas*): Local adaptations to different tidal environments? Limnology and Oceanography 59:588-602.

Moksnes, P. O., P. R. Jonsson, M. Nilsson Jacobi, and K. Vikström. 2014b. Larval connectivity and ecological coherence of marine protected areas (MPAs) in the Kattegat-Skagerrak region. Swedish Institute for the Marine Environment. Report.

Moland, E., E. Moland Olsen, H. Knutsen, J. A. Knutsen, S. E. Enersen, C. André, and N. C. Stenseth. 2011a. Activity patterns of wild European lobster *Homarus gammarus* in coastal marine reserves: implications for future reserve design. Marine Ecology Progress Series 429:197-207.

Moland, E., E. M. Olsen, K. Andvord, J. A. Knutsen, and N. C. Stenseth. 2011b. Home range of European lobster (*Homarus gammarus*) in a marine reserve: implications for future reserve design. Canadian Journal of Fisheries and Aquatic Sciences 68:1197-1210.

Moland, E., E. M. Olsen, H. Knutsen, P. Garrigou, S. H. Espeland, A. R. Kleiven, C. André, and J. A. Knutsen. 2013a. Lobster and cod benefit from small-scale northern marine protected areas: inference from an empirical before–after control-impact study. Proceedings of the Royal Society B: Biological Sciences 280:1754.

Moland, E., M. Ulmestrand, E. M. Olsen, and N. C. Stenseth. 2013b. Long-term decrease in sex-specific natural mortality of European lobster within a marine protected area. Marine Ecology Progress Series 491:153-164.

Momigliano, P., G. P. J. Denys, H. Jokinen, and J. Merilä. 2018. *Platichthys solemdali* sp. nov. (Actinopterygii, Pleuronectiformes): A new flounder species from the Baltic Sea. Frontiers in Marine Science 5:225.

Momigliano, P., H. Jokinen, A. Fraimout, A.-B. Florin, A. Norkko, and J. Merilä. 2017. Extraordinarily rapid speciation in a marine fish. Proceedings of the National Academy of Sciences 114:6074-6079.

Möller, S., H. M. Winkler, S. Richter, and R. Bastrop. 2021. Genetic population structure of pike (*Esox lucius* Linnaeus, 1758) in the brackish lagoons of the southern Baltic Sea. Ecology of Freshwater Fish 30:140-149.

Nielsen, E. E., P. Grønkjær, D. Meldrup, and H. Paulsen. 2005. Retention of juveniles within a hybrid zone between North Sea and Baltic Sea Atlantic cod (*Gadus morhua*). Canadian Journal of Fisheries and Aquatic Sciences 62:2219-2225.

Nielsen, E. E., M. M. Hansen, D. E. Ruzzante, D. Meldrup, and P. Grønkjær. 2003. Evidence of a hybrid-zone in Atlantic cod (*Gadus morhua*) in the Baltic and the Danish Belt Sea revealed by individual admixture analysis. Molecular Ecology 12:1497-1508.

Nielsen, E. E., P. H. Nielsen, D. Meldrup, and M. M. Hansen. 2004. Genetic population structure of turbot (*Scophthalmus maximus* L.) supports the presence of multiple hybrid zones for marine fishes in the transition zone between the Baltic Sea and the North Sea. Molecular Ecology 13:585-595.

Nordahl, O., P. Koch-Schmidt, J. Sunde, Y. Yıldırım, P. Tibblin, A. Forsman, and P. Larsson. 2019. Genetic differentiation between and within ecotypes of pike (Esox lucius) in the Baltic Sea. Aquatic Conservation: Marine and Freshwater Ecosystems 29:1923-1935.

Olsson, J., A.-B. Florin, K. Mo, T. Aho, and N. Ryman. 2012. Genetic structure of whitefish (*Coregonus maraena*) in the Baltic Sea. Estuarine, Coastal and Shelf Science 97:104-113.

Olsson, J., K. Mo, A. B. Florin, T. Aho, and N. Ryman. 2011. Genetic population structure of perch *Perca fluviatilis* along the Swedish coast of the Baltic Sea. Journal of Fish Biology 79:122-137.

Orth, R. J., M. Luckenbach, and K. A. Moore. 1994. Seed dispersal in a marine macrophyte: implications for colonization and restoration. Ecology 75:1927-1939.

Palm, S., J. Dannewitz, T. Järvi, M.-L. Koljonen, T. Prestegaard, and K. H. Olsén. 2008. No indications of Atlantic salmon (*Salmo salar*) shoaling with kin in the Baltic Sea. Canadian Journal of Fisheries and Aquatic Sciences 65:1738-1748.

Palmé, A., L. Wennerström, P. Guban, N. Ryman, and L. Laikre. 2012. Compromising Baltic salmon genetic diversity - conservation genetic risks associated with compensatory releases of salmon in the Baltic Sea

Panova, M., J. Hollander, and K. Johannesson. 2006. Site-specific genetic divergence in parallel hybrid zones suggests nonallopatric evolution of reproductive barriers. Molecular Ecology 15:4021-4031.

Pedersen, S., G. Rasmussen, E. E. Nielsen, L. Karlsson, and P. Nyberg. 2007. Straying of Atlantic salmon, *Salmo salar*, from delayed and coastal releases in the Baltic Sea, with special focus on the Swedish west coast. Fisheries Management and Ecology 14:21-32.

Pereyra, R. T., C. Huenchuñir, D. Johansson, H. Forslund, L. Kautsky, P. R. Jonsson, and K. Johannesson. 2013. Parallel speciation or long-distance dispersal? Lessons from seaweeds (Fucus) in the Baltic Sea. Journal of Evolutionary Biology 26:1727-1737.

Reis-Santos, P., S. E. Tanner, M. A. Aboim, R. P. Vasconcelos, J. Laroche, G. Charrier, M. Pérez, P. Presa, B. M. Gillanders, and H. N. Cabral. 2018. Reconciling differences in natural tags to infer demographic and genetic connectivity in marine fish populations. Scientific Reports 8:10343.

Reusch, T. B. H. 2002. Microsatellites reveal high population connectivity in eelgrass (*Zostera marina*) in two contrasting coastal areas. Limnology and Oceanography 47:78-85.

Rohtla, M., R. Svirgsden, I. Taal, L. Saks, R. Eschbaum, and M. Vetemaa. 2015. Life-history characteristics of ide *Leuciscus idus* in the Eastern Baltic Sea. Fisheries Management and Ecology 22:239-248.

Rothäusler, E., H. Corell, and V. Jormalainen. 2015. Abundance and dispersal trajectories of floating Fucus vesiculosus in the Northern Baltic Sea. Limnology and Oceanography 60:2173-2184.

Saulamo, K., and E. Neuman. 2002. Local management of Baltic fish stocks - significance of migrations. Fiskeriverket, Finfo 2002:9, 1-18.

Saulamo, K., and G. Thoresson. 2005. Management of pikeperch migrating over management areas in a Baltic Archipelago area. AMBIO: A Journal of the Human Environment 34:120-124.

Sayer, M. D. J. 2001. Duration of refuge residence by goldsinny, *Ctenolabrus rupestris*. Journal of the Marine Biological Association of the United Kingdom 79:571-572.

Sefbom, J., A. Kremp, K. Rengefors, P. R. Jonsson, C. Sjöqvist, and A. Godhe. 2018. A planktonic diatom displays genetic structure over small spatial scales. Environmental Microbiology 20:2783-2795.

Spies, I., L. Hauser, P. E. Jorde, H. Knutsen, A. E. Punt, L. A. Rogers, and N. C. Stenseth. 2018. Inferring genetic connectivity in real populations, exemplified by coastal and oceanic Atlantic cod. Proceedings of the National Academy of Sciences 115:4945-4950.

Stakėnas, S., and A. Pilinkovskij. 2019. Migration patterns and survival of stocked Atlantic sturgeon (*Acipenser oxyrinchus* Mitchill, 1815) in Nemunas Basin, Baltic Sea. Journal of Applied Ichthyology 35:128-137.

Strelkov, P., M. Katolikova, and R. Väinolä. 2017. Temporal change of the Baltic Sea–North Sea blue mussel hybrid zone over two decades. Marine Biology 164:214.

Stroganov, A. N., M. Bleil, R. Oeberst, H. Winkler, and A. V. Semenova. 2013. On differentiation of cod (*Gadus morhua* L.) groups in Baltic Sea. Russian Journal of Genetics 49:937-944.

Stuckas, H., L. Knöbel, H. Schade, C. Breusing, H. H. Hinrichsen, M. Bartel, K. Langguth, and F. Melzner. 2017. Combining hydrodynamic modelling with genetics: can passive larval drift shape the genetic structure of Baltic Mytilus populations? Molecular Ecology 26:2765-2782.

Svedäng, H., J. M. I. Barth, A. Svenson, P. Jonsson, S. Jentoft, H. Knutsen, and C. André. 2018. Local cod (*Gadus morhua*) revealed by egg surveys and population genetic analysis after longstanding depletion on the Swedish Skagerrak coast. ICES Journal of Marine Science 76:418-429.

Symonds, D. J., and S. I. Rogers. 1995. The influence of spawning and nursery grounds on the distribution of sole Solea solea (L.) in the Irish Sea, Bristol Channel and adjacent areas. Journal of Experimental Marine Biology and Ecology 190:243-261.

Säisä, M., M. Salminen, M.-L. Koljonen, and J. Ruuhijärvi. 2010. Coastal and freshwater pikeperch (*Sander lucioperca*) populations differ genetically in the Baltic Sea basin. Hereditas 147:205-214.

Tatarenkov, A., R. B. Jönsson, L. Kautsky, and K. Johannesson. 2007. Genetic structure in populations of Fucus vesiculosus (Phaeophyceae) over spatial scales from 10 m to 800 km. Journal of Phycology 43:675-685.

Teacher, A. G., C. André, P. R. Jonsson, and J. Merilä. 2013. Oceanographic connectivity and environmental correlates of genetic structuring in Atlantic herring in the Baltic Sea. Evolutionary Applications 6:549-567.

Thorbjørnsen, S. H., E. Moland, M. B. O. Huserbråten, J. A. Knutsen, H. Knutsen, and E. M. Olsen. 2018. Replicated marine protected areas (MPAs) support movement of larger, but not more, European lobsters to neighbouring fished areas. Marine Ecology Progress Series 595:123-133.

Tibblin, P., A. Forsman, T. Borger, and P. Larsson. 2016. Causes and consequences of repeatability, flexibility and individual fine-tuning of migratory timing in pike. Journal of Animal Ecology 85:136-145.

Tonteri, A., A. J. Veselov, S. Titov, J. Lumme, and C. R. Primmer. 2007. The effect of migratory behaviour on genetic diversity and population divergence: a comparison of anadromous and freshwater Atlantic salmon Salmo salar. Journal of Fish Biology 70:381-398.

Triest, L., and T. Sierens. 2015. Strong bottlenecks, inbreeding and multiple hybridization of threatened European *Ruppia maritima* populations. Aquatic Botany 125:31-43.

Triest, L., T. Sierens, D. Menemenlis, and T. Van der Stocken. 2018. Inferring connectivity range in submerged aquatic populations (Ruppia L.) along European coastal lagoons from genetic imprint and simulated dispersal trajectories. Frontiers in Plant Science 9:806.

Ulrich, C., J. Hemmer-Hansen, J. Boje, A. Christensen, K. Hüssy, H. Sun, and L. W. Clausen. 2017. Variability and connectivity of plaice populations from the Eastern North Sea to the Baltic Sea, part II. Biological evidence of population mixing. Journal of Sea Research 120:13-23.

Ungfors, A., H. Hallbäck, and P. G. Nilsson. 2007. Movement of adult edible crab (*Cancer pagurus* L.) at the Swedish West Coast by mark-recapture and acoustic tracking. Fisheries Research 84:345-357.

Ungfors, A., N. J. McKeown, P. W. Shaw, and C. André. 2009. Lack of spatial genetic variation in the edible crab (*Cancer pagurus*) in the Kattegat–Skagerrak area. ICES Journal of Marine Science 66:462-469.

Weber, W. 1975. A tagging experiment on spring-spawning herring of the Kiel Bay. Berichte der Deutschen Wissenschaftlichen Kommission fuer Meeresforschung 24:184-188.

Wennerström, L., J. Olsson, N. Ryman, and L. Laikre. 2016. Temporally stable, weak genetic structuring in brackish water northern pike (*Esox lucius*) in the Baltic Sea indicates a contrasting divergence pattern relative to freshwater populations. Canadian Journal of Fisheries and Aquatic Sciences 74:562-571.

Westgaard, J.-I., A. Staby, J. Aanestad Godiksen, A. J. Geffen, A. Svensson, G. Charrier, H. Svedäng, and C. André. 2017. Large and fine scale population structure in European hake (*Merluccius merluccius*) in the Northeast Atlantic. ICES Journal of Marine Science 74:1300-1310.

Whitlock, R., S. Mäntyniemi, S. Palm, M.-L. Koljonen, J. Dannewitz, and J. Östergren. 2018. Integrating genetic analysis of mixed populations with a spatially explicit population dynamics model. Methods in Ecology and Evolution 9:1017-1035.

Whittaker, B. A., S. Consuegra, and C. Garcia de Leaniz. 2018. Genetic and phenotypic differentiation of lumpfish (*Cyclopterus lumpus*) across the North Atlantic: implications for conservation and aquaculture. PeerJ 6:e5974.

Villegas-Ríos, D., J. Alós, D. March, M. Palmer, G. Mucientes, and F. Saborido-Rey. 2013. Home range and diel behavior of the ballan wrasse, Labrus bergylta, determined by acoustic telemetry. Journal of Sea Research 80:61-71.

Øresland, V., and C. André. 2008. Larval group differentiation in Atlantic cod (*Gadus morhua*) inside and outside the Gullmar Fjord. Fisheries Research 90:9-16.

Øresland, V., and M. Ulmestrand. 2013. European lobster subpopulations from limited adult movements and larval retention. ICES Journal of Marine Science 70:532-539.

Östergren, J., and J. Nilsson. 2012. Importance of life-history and landscape characteristics for genetic structure and genetic diversity of brown trout (*Salmo trutta* L.). Ecology of Freshwater Fish 21:119-133.

Östergren, J., J. Nilsson, and H. Lundqvist. 2012. Linking genetic assignment tests with telemetry enhances understanding of spawning migration and homing in sea trout *Salmo trutta* L. Hydrobiologia 691:123-134.
